# Perceiving molecular evolution processes in *Escherichia coli *by comprehensive metabolite and gene expression profiling

**DOI:** 10.1186/gb-2008-9-4-r72

**Published:** 2008-04-10

**Authors:** Chandran Vijayendran, Aiko Barsch, Karl Friehs, Karsten Niehaus, Anke Becker, Erwin Flaschel

**Affiliations:** 1 International NRW Graduate School in Bioinformatics and Genome Research, Bielefeld University, D-33594 Bielefeld, Germany; 2 Fermentation Engineering Group, Bielefeld University, D-33594 Bielefeld, Germany; 3 Faculty of Biology, Bielefeld University, D-33594 Bielefeld, Germany

## Abstract

**Background:**

Evolutionary changes that are due to different environmental conditions can be examined based on the various molecular aspects that constitute a cell, namely transcript, protein, or metabolite abundance. We analyzed changes in transcript and metabolite abundance in evolved and ancestor strains in three different evolutionary conditions - excess nutrient adaptation, prolonged stationary phase adaptation, and adaptation because of environmental shift - in two different strains of bacterium *Escherichia coli *K-12 (MG1655 and DH10B).

**Results:**

Metabolite profiling of 84 identified metabolites revealed that most of the metabolites involved in the tricarboxylic acid cycle and nucleotide metabolism were altered in both of the excess nutrient evolved lines. Gene expression profiling using whole genome microarray with 4,288 open reading frames revealed over-representation of the transport functional category in all evolved lines. Excess nutrient adapted lines were found to exhibit greater degrees of positive correlation, indicating parallelism between ancestor and evolved lines, when compared with prolonged stationary phase adapted lines. Gene-metabolite correlation network analysis revealed over-representation of membrane-associated functional categories. Proteome analysis revealed the major role played by outer membrane proteins in adaptive evolution. GltB, LamB and YaeT proteins in excess nutrient lines, and FepA, CirA, OmpC and OmpA in prolonged stationary phase lines were found to be differentially over-expressed.

**Conclusion:**

In summary, we report the vital involvement of energy metabolism and membrane-associated functional categories in all of the evolutionary conditions examined in this study within the context of transcript, outer membrane protein, and metabolite levels. These initial data obtained may help to enhance our understanding of the evolutionary process from a systems biology perspective.

## Abstract 2

Transcript and metabolite abundance changes were analyzed in evolved and ancestor strains of *Escherichia coli* in three different evolutionary conditions

## Background

Most micro-organisms grow in environments that are not favorable for their growth. The level of nutrients available to them is rarely optimal. These microbes must adapt to environmental conditions that consist of excess, suboptimal (limiting) or fluctuating levels of nutrients, or famine. Evolution can be studied by observing its processes and consequences in the laboratory, specifically by culturing a micro-organism in varying nutrient environments [1-4]. Extensively studied microbial evolutionary processes include nutrient-limited adaptive evolution [5-7] and famine-induced prolonged stationary phase evolution [8-10]. During prolonged carbon starvation, micro-organisms can undergo rapid evolution, with mutants exhibiting a 'growth advantage in stationary phase' (GASP) phenotype [2]. These mutants, harboring a selective advantage, out-compete their siblings and take over the culture through their progeny [11-13]. Adaptive evolution of micro-organisms is a process in which specific mutations result in phenotypic attributes that are responsible for fitness in a particular selective environment [1]. Laboratory studies conducted under these evolutionary conditions can address fundamental questions regarding adaptation processes and selection pressures, thereby explaining modes of evolution.

In this study we used *Escherichia coli *K-12 strains (MG1655 and DH10B) subjected to the following processes: a serial passage system (excess nutrient adaptive evolution studies), constant batch culture (prolonged stationary phase evolution studies), and culture with nutrient alteration after adaptation to a particular nutrient (examining pleiotropic effects due to environmental shift). During adverse conditions, micro-organisms are known to exploit limited resources more quickly and are observed to assimilate various metabolites. Some of these residual metabolites comprise an alternative resource that the organism can metabolize [2]. Continual assimilation of metabolites and the various compounds metabolized by the organism offer a specific niche that allows the organism to evolve with genetic capacity to utilize those assimilated metabolites [2]. Hence, a detailed metabolite analysis of these evolved populations would enhance our understanding of these evolutionary processes. Along with data generated from transcriptomics approaches, metabolomics data will be vital in obtaining a global view of an organism at a particular time point, during which metabolite behavior closely reflects the actual cellular environment and the observed phenotype of that organism.

We applied metabolome and gene expression profiling approaches to elucidate excess nutrient adaptive evolution, prolonged stationary phase evolution, and pleiotropic effects due to environmental shift in two strains of differing genotype. To eliminate the possibility of the strain-dependent phenomenon of evolution and to examine the parallelism of the laboratory evolution process, we examined in two strains the evolutionary processes referred to above. Hence, the groups in which we compared the metabolite and gene expression profiles were as follows (Table 1): MG and DH (MG1655 and DH10B *E. coli *strains grown in glucose, respectively); MGGal and DHGal (MG1655 and DH10B grown in galactose); MGAdp and DHAdp (MG1655 and DH10B adapted about 1,000 generations in glucose); MGAdpGal and DHAdpGal (MGAdp and DHAdp [the glucose evolved strains] grown in galactose); and MGStat and DHStat (MG1655 and DH10B grown in prolonged stationary phase; 37 days).

**Table 1:** Strains and their evolved conditions

Strain abbreviations	Evolved condition
MG	MG1655 grown in glucose (ancestor)
DH	DH10B grown in glucose (ancestor)
MGGal	MG1655 grown in galactose (ancestor)
DHGal	DH10B grown in galactose (ancestor)
MGAdp	MG1655 adapted about 1,000 generations in glucose (evolved)
DHAdp	DH10B adapted about 1,000 generations in glucose (evolved)
MGAdpGal	MGAdp (glucose evolved strains) grown in galactose (evolved)
DHAdpGal	DHAdp (glucose evolved strains) grown in galactose (evolved)
MGStat	MG1655 grown in prolonged stationary phase (37 days; evolved)
DHStat	DH10B grown in prolonged stationary phase (37 days; evolved)

In this study we developed a picture of laboratory molecular evolutionary processes in two different strains by integrating multidimensional metabolome and gene expression data, in order to identify metabolites and genes that are vital to the evolutionary process.

## Results

The Adp line cultures (MGAdp and DHAdp) were maintained in prolonged exponential growth phase by daily passage into fresh medium for about 1,000 generations, undergoing many rounds of exponential phase growth. The Stat line cultures (MGStat and DHStat) were maintained in constant batch culture for 37 days, during which no nutrients were added after the initial inoculation and no cells were removed (unlike the preceding setup). For the AdpGal line cultures (MGAdpGal and DHAdpGal), Adp lines (glucose adapted) were grown in medium containing galactose as carbon source, thus creating an environmental shift for the cells with respect to the standard nutrient source. During this period of adaptation, both Adp lines (evolved) exhibited increased fitness in their growth, whereas Stat lines (evolved) exhibited growth behavior similar to that of their ancestors. The samples of MG, DH, MGGal, DHGal, MGAdp, DHAdp, MGAdpGal, DHAdpGal, MGStat, and DHStat lines grown in the respective carbon sources (Table 1) were harvested during the mid-exponential phase of growth for both metabolome and transcriptome analysis.

In the metabolome analysis, from about 200 peaks in each chromatogram about 100 metabolites were identified by gas chromatography-mass spectrometry. In the transcriptome analysis a whole genome microarray consisting of 4,288 open reading frames of *Escherichia coli *K-12 was used. To examine the multivariate measures of variability of the metabolite and gene expression profiles for the obtained data, and for clustering the biological samples, we applied principal components analysis (PCA). In order to identify parallel metabolite accumulation and gene expression, we applied pair-wise correlation plot analysis. To examine the extent of parallelism among the evolved lines, gene-metabolite correlation networks were constructed and their topologic properties were studied. By mapping the correlation networks to Gene Ontology (GO) functional annotations, the functional relevance of the networks was determined. Subsequently, the functional modules that were statistically significantly over-represented in respective evolution processes were identified.

### Metabolome profiling

**Figure 1:**
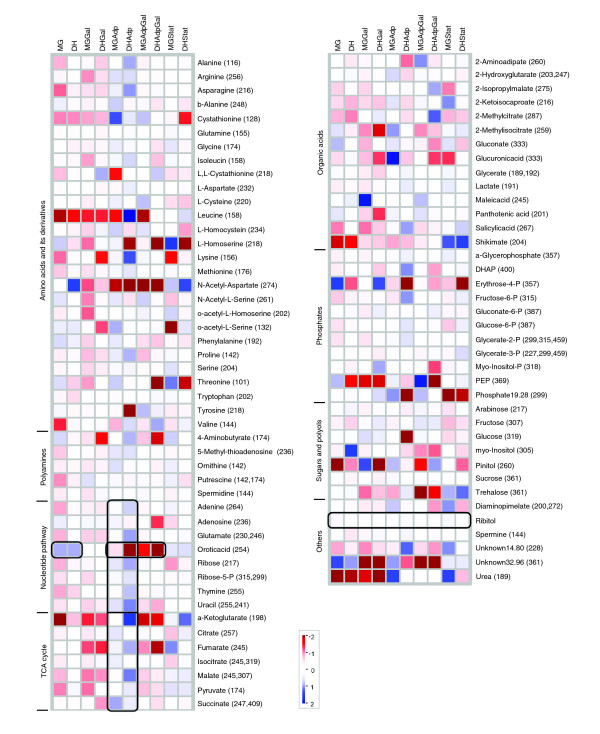
Overlay heat map of the metabolite profiles. Logarithmically transformed (to base 2) averaged absolute values were used to plot the heat map. Red or blue color indicates that the metabolite content is decreased or increased, respectively. For each sample, gas chromatography/mass spectrometry was used to quantify 84 metabolites (nonredundant), categorized into amino acids and their derivatives, polyamines, metabolites involved in nucleotide related pathways, tricarboxylic acid (TCA) cycle, organic acids, phosphates, and sugar and polyols. The m/z values given for each metabolite in parentheses are the selective ions used for quantification. Highlighted black boxes indicate significant changes in the metabolite level in the TCA cycle and the nucleotide related pathways of the evolved lines. The internal standard ribitol metabolite level is also highlighted, which is shown as control.

**Figure 2:**
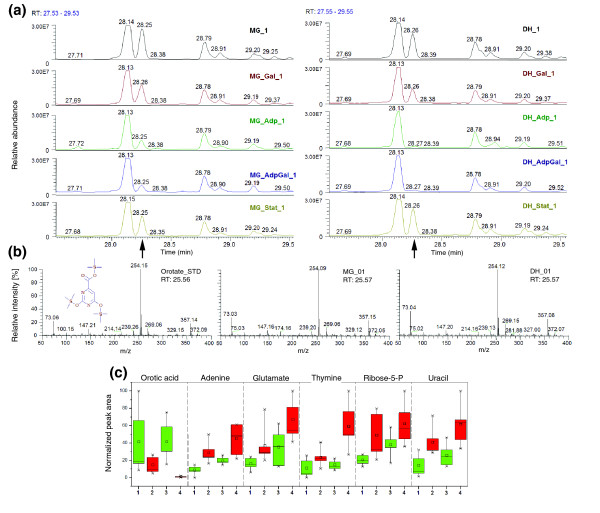
Typical examples of metabolite differential levels among the ancestral and evolved lines. **(a) **Sections of chromatograms showing orotate or orotic acid (denoted by an arrow) abundance among all the lines. **(b) **Mass spectrum of orotate purified standard and mass spectrum of the identified peak as orotate in both strains. **(c) **Box and Whisker plots of metabolites involved in nucleotide related pathways. 1 and 3 represent MG and DH lines (ancestors); 2 and 4 represent MGAdp and DHAdp lines (evolved). The top and bottom of each box represent the 25th and 75th percentiles, the centre square indicates the mean, and the extents of the whiskers show the extent of the data. For each metabolite, the maximal measured peak area was normalized to a value of 100.

Metabolome profiling has frequently been applied to obtain quantitative information on metabolites for studies on mutational [14] or environmental effects [15], but not in an evolutionary context. Here, for our evolutionary studies, we used an approach that combined metabolomics and transcriptomics that offers whole genome coverage. In total, 84 metabolites of known chemical structure were quantified in every chromatogram (see Additional data file 1). The full datasets from the metabolite profiling study are presented in an overlay heat map (Figure 1). This map shows the averaged absolute values of all indentified metabolites of the samples analyzed. In most cases the levels of metabolites are significantly changed in evolved lines, and their directional behavior is more or less constant in both the ancestral strains and in their evolved strains (Figure 2).

In the comparison between MGAdp and DHAdp strains, out of 111 metabolites 50% (55 metabolites) and 55% (61 metabolites) of them had score *d*_*i *_≥ 1 or ≤ -1 (significance analysis of microarrays [SAM], *T *statistic value) [16], of which 27% (31) of metabolites were common to both strains. The MGAdpGal and DHAdpGal strains were observed to have 39% (43 metabolites) and 33% (37 metabolites), respectively, where 13% (10) of the metabolites were common to both of these strains. Likewise, MGStat and DHStat exhibited differences in 48% (53 metabolites) and 37% (41 metabolites) of the cases, and 20% (19) of metabolites were common in both strains (Table 2; also see Additional data file 2).

**Table 2:** Statistically significant metabolites involved in various evolved conditions

Evolved condition	Total number of metabolites taken into account	Number of over-abundant metabolites (*d*_*i *_≥ 1)	Number of less abundant metabolites (*d*_*i *_≤ -1)	Total number of differentially abundant metabolites	Number of intersecting metabolites	Total number of intersecting metabolites
MGAdp	111	48	7	55	27 (+)	31
DHAdp	111	39	22	61	4 (-)	
MGAdpGal	111	37	6	43	7 (+)	10
DHAdpGal	111	18	19	37	3 (-)	
MGStat	111	36	17	53	12 (+)	19
DHStat	111	20	21	41	7 (-)	

Metabolites were assumed to be significant when their score *d*_*i *_≥ 1 or ≤ -1 (significance analysis of microarrays, *T *statistic value). (+), over-abundant/expressed candidates; (-), less abundant/under-expressed candidates.

**Figure 3:**
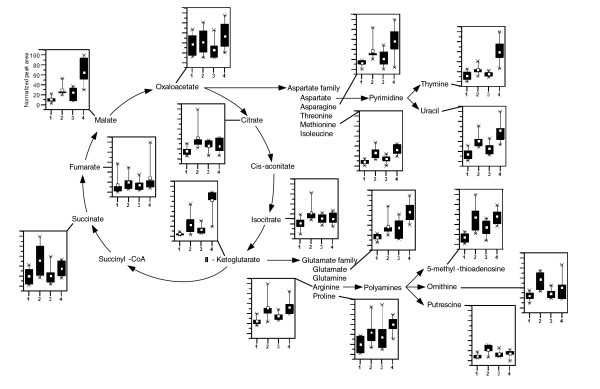
Levels of metabolites involved in TCA cycle and diversion of key intermediates to biosynthetic pathways. In the box and whisker plots, 1 and 3 represent MG and DH lines (ancestors), and 2 and 4 represent MGAdp and DHAdp lines (evolved). The top and bottom of each box represent the 25th and 75th percentiles, the centre square indicates the mean, and the extents of the whiskers show the extent of the data. For each metabolite, the maximal measured peak area was normalized to a value of 100.

Those metabolites that exhibited differences between ancestral and evolved strains fell into groups of metabolites involved in tricarboxylic acid (TCA) cycle, nucleotide metabolism, amino acids and their derivatives, and polyamine biosynthesis (Figure 1). For example, metabolites that are involved in the nucleotide pathway were significantly different between both ancestral and evolved strains (MG/MGAdp: *P*= 0.007; DH/DHAdp: *P *= 0.038 [Wilcoxon rank sum test; Benjamini-Hochberg corrected; a false discovery rate-controlled *P*-value cutoff of ≤ 0.05]). Nucleic acids - adenine, thymine and uracil - along with ribose-5-phosphate and orotate (orotic acid) metabolite levels significantly differed in both of the Adp evolved strains (Figure 2c). Orotate is an intermediate in *de novo *biosynthesis of pyrimidine ribonucleotides, levels of which were high in ancestor strains, which was not the case for other metabolites that were not intermediates in this process (Figure 2a, b, c). Likewise, levels of metabolites involved in the TCA cycle were significantly different for both ancestral and evolved strains (MG/MGAdp: *P *= 3.70 × e^-06^; DH/DHAdp: *P *= 0.026 [Wilcoxon rank sum test; Benjamini-Hochberg corrected; a false discovery rate-controlled *P*-value cutoff of ≤ 0.05]). An overview of the TCA cycle and the diversion of its key intermediates reveal clear differences in metabolite levels among the Adp evolved strains and their ancestors in both strains (Figure 3). Because the TCA cycle is the first step in generating precursors for various biosynthesetic processes and is among the main energy-producing pathways in a cell, changes in these metabolite levels can be expected to play a vital role in the adaptive evolution of these evolved strains, which exhibited increased fitness in growth compared with their ancestor strains.

### Gene expression profiling

Several studies have used gene expression profiling to study molecular evolution, but these studies were confined to a single type of evolutionary process and were focused on a single molecular aspect that characterizes a cell (transcript abundance) [17-20]. In our study we focused on three evolutionary conditions in two strains and two molecular aspects of a cell (transcript and metabolite abundance). This approach allowed us to integrate metabolome and transcriptome datasets to elucidate the process of adaptive evolution under laboratory conditions.

**Figure 4:**
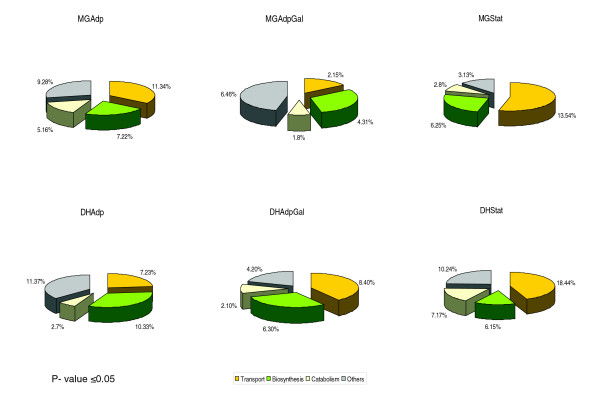
Broad functional annotations of the transcriptome profiling data. The pie charts of individual evolutionary experimental conditions show the distribution of differentially regulated Gene Ontology (GO) functional modules consisting various functional categories, having *P *≤ 0.05 (Wilcoxon rank sum test). The values represent the number of GO functional categories associated with that GO functional module. For each evolutionary condition the details of GO functional modules and its significant values are provided in Additional data file 4.

Using the whole genome microarray, consisting of 4,288 open reading frames, we compared expression levels of the transcripts in all of the evolved conditions. The comparison of MG/MGAdp and DH/DHAdp lines among 4,159 genes revealed that 15% (633 genes) and 19% (814 genes), respectively, had altered expression levels (score *d*_*i *_≥ 1 or ≤ -1; SAM, *T*-statistic value [16]). Among these, 18% (263) of the genes were common to both strains. In the MGGal/MGAdpGal versus DHGal/DHAdpGal comparison of 4,126 genes, we observed there to be a 5% (206 genes) and 16% (674 genes) change, respectively, and 4% (35 genes) of these genes were common to both strains. Likewise, on comparing MG/MGStat versus DH/DHStat, we observed that 14% (569 genes) and 20% (825 genes) of the 4,156 genes had altered expression levels, of which 9% (120 genes) were common to both strains (Table 3; also see Additional data file 3). In all comparisons, statistically significant functional categories (with *P *≤ 0.05 [Wilcoxon rank sum test]) that did exhibit differences between ancestral and the evolved strains fell into broad groups of genes that are involved in transport, biosynthesis, and catabolism (Figure 4). The gene expression changes associated with these main and broad functional categories consist of groups emphasizing specific functions (see Additional data file 4). For example, genes involved in the pentose phosphate pathway were significantly differentially expressed between ancestral and evolved strains of the Adp lines (MG/MGAdp: *P *= 0.036; DH/DHAdp: *P *= 0.019; see Additional data files 5 and 6). The pentose phosphate pathway produces the precursors (pentose phosphates) for ribose and deoxyribose in the nucleic acids. The accumulation of nucleic acid metabolites (Figures 1 and 2) and over-expression of pentose phosphate pathway genes in the Adp lines allow us to assume that the pentose phosphate pathway is involved in adaptive evolution occurring in response to excess nutrient.

**Table 3:** Statistically significant genes involved in various evolved conditions

Evolved condition	Total number of genes taken into account	Number of over-expressed genes (*d*_*i *_≥ 1)	Number of under-expressed genes (*d*_*i *_≤ -1)	Total number of differentially expressed genes	Number of intersecting genes	Total number of intersecting genes
MGAdp	4,159	315	318	633	116 (+)	263
DHAdp	4,159	438	376	814	147 (-)	
MGAdpGal	4,126	91	115	206	5 (+)	35
DHAdpGal	4,126	357	317	674	30 (-)	
MGStat	4,156	306	263	569	69 (+)	120
DHStat	4,156	452	373	825	51 (-)	

Genes were assumed to be significant when their score *d*_*i *_≥ 1 or ≤ -1 (significance analysis of microarrays, *T *statistic value). (+), over-abundant/expressed candidates; (-), less abundant/under-expressed candidates.

### Extent of changes

**Figure 5:**
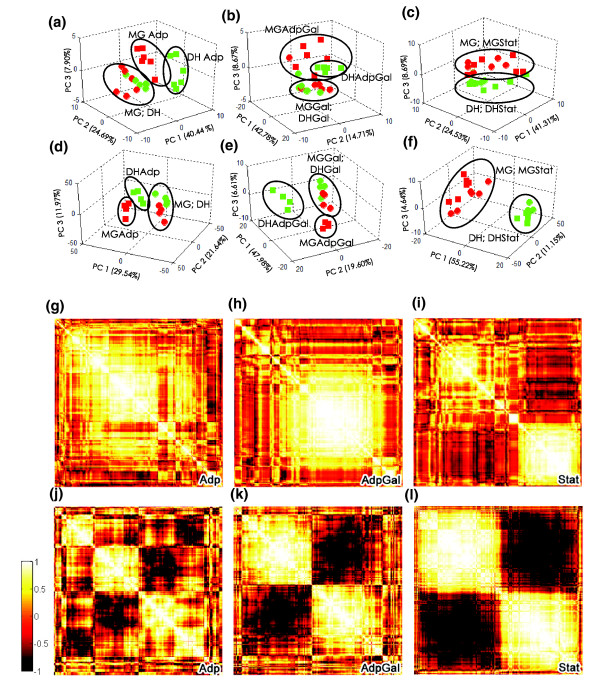
The extent of changes in experimental evolution among the strains. **(a-f) **Principal components analysis (PCA) of the metabolome (panels a to c) and transcriptome (panels d to f) data; each data point represents an experimental sample plotted using the first three principal components. PCA was carried out on the log-transformed mean-centred data matrix using all identified metabolites and the genes with *P *≤ 0.05 (Student's *t*-test) in at least one strain. Values given for each component in parentheses represents the percentage of variance. **(g-l) **Pair-wise correlation maps of the metabolome (panels g to i) and transcriptome (panels j to l) data among the strains, using Pearson correlation coefficient (*r*). All of the metabolites and the genes having a threshold value of *r *≤ -0.9 or ≥ 0.9 were plotted and color coded on both axes of a matrix containing all pair-wise metabolite or gene expression profile correlation. Darker spots indicate greater degrees of negative correlation among the strains. Both the analyses were carried out using Matlab 6.5 (The MathWorks, Inc., Natick, MA, USA).

To examine the level of metabolite and gene expression changes among all the evolutionary conditions, we applied PCA, which is a technique for conducted multivariate data analysis that reduces the dimensionality and complexity of the dataset without losing the ability to calculate accurate distance metrics. It transforms the metabolome and transcript expression data into a more manageable form, in which the number of clusters might be discriminated. When applied to ancestor and Adp lines, both ancestors (MG and DH) cluster together; Adp lines (MGAdp and DHAdp) cluster separately from their ancestor lines, denoting substantial adaptive changes. This pattern was observed in both the metabolite and gene expression data, as summarized in Figure 5a, d. When PCA was applied to MGGal, DHGal and AdpGal lines, the MGGal and DHGal lines clustered together; AdpGal lines clustered separately from their ancestor lines, denoting considerable pleiotropic changes due to environmental shift in both metabolite and gene expression data (Figure 5b, e). Unlike Adp and AdpGal lines, Stat lines exhibited dissimilar behaviors; Stat lines (MGStat and DHStat) clustered along with their ancestor lines (MG and DH), denoting few changes between ancestor and evolved strains or diverse changes between the evolved strains in both metabolite and gene expression data (Figure 5c, f). To determine the extent of adaptation in these evolved lines, we examined whether the media was the greatest determination of variance or whether the adaptation was greater. To this end, we conducted PCA analyses for both the ancestors and evolved lines of both the strains grown in two different media (MG, MGAdp, DH, DHAdp, MGGal, MGAdpGal, DHGal, and DHAdGal). Both the ancestor strains grown in different media clustered together, and both evolved strains grown in different medium clustered together; this suggests that adaption was the greatest determinant of variance (see Additional data file 7).

### Direction of the observed extent of changes

To examine the level of observed change among the strains, we calculated the pair-wise Pearson correlation coefficient (*r*; PCC) for all of the metabolites and significantly correlating genes. All genes having a threshold of *r *≤ -0.9 or ≥ 0.9 and all metabolites were plotted on both axes of a matrix containing either all pair-wise metabolite or gene expression profile correlations. When these correlations (*r*) are color coded, this facilitates use of visual inspection to determine the degree of positive and negative correlation among the samples in question. The correlation map of Adp, AdpGal, and Stat line comparisons exhibited various degrees of negative correlation (Figure 5g-l). Among these, Stat line comparisons (MG/MGStat versus DH/DHStat) exhibited a high degree of negative correlation when compared with AdpGal and Adp line comparisons in both metabolite and gene expression correlation maps (Fig. 5i, l), suggesting elevated levels of variability due to selection among the Stat lines. The correlation map of the Adp line comparison (MG/MGAdp versus DH/DHAdp) revealed a lower degree of negative correlation than did the other line comparisons in both metabolite and gene expression correlation maps (Figure 5g, j), denoting a reduced level of variability caused by selection among the Adp lines.

### Gene-metabolite correlation network analysis

**Figure 6:**
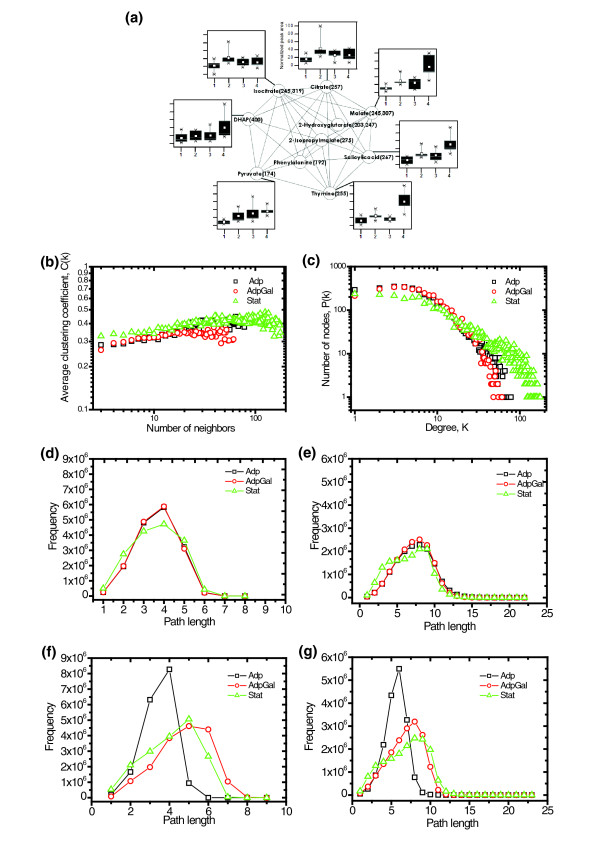
Gene-to-metabolite correlation network analyses. **(a) **Substructure extracted from Adp correlation network with MCODE algorithm, showing preferentially linked functionally related metabolites. The m/z values of selective ions used for quantification are shown in parentheses for each metabolite. In the box and whisker plots of the metabolites 1 and 3 represent MG and DH lines (ancestors), and 2 and 4 represent MGAdp and DHAdp lines (evolved). **(b-g) **Topologic properties of all evolution-specific coexpression networks. Panel b shows the degree distribution of the clustering coefficients of all of the evolution-specific network entities. The average clustering coefficient of all the nodes was plotted against the number of neighbours. Panel c shows the degree distribution of the networks; the number of nodes with a given degree (*k*) in the networks approximates a power law (P [*k*] about *k*^γ ^; Adp γ = 1.70, AdpGal γ = 1.76, and Stat γ = 1.32). Distribution of the shortest path between pairs of nodes in the evolution specific (panels d and e) and intersection (panels f and g) networks; constructed with principal components analysis thresholds of 0.8 (panels d and f) and 0.9 (panels e and g).

It has been demonstrated that functionally related genes are preferentially linked in co-expression networks [21]. By integrating and comparing the gene expression and metabolite profile patterns, we were able to explore the connections between the gene-gene and gene-metabolite links and associated functions (Figure 6a) by assuming that the more similar the expression pattern is, the shorter is the distance between genes and/or metabolites in the co-expression network. Relative transcript amounts of all genes and relative concentrations of all nonredundant metabolites were combined to form distance matrices, which were calculated by using the PCC to build co-expression networks. In many cases there were striking relationships between network substructure, gene, or metabolite function and co-expression (Figure 6a). The co-expression network analysis provides a possibility to use it as a quantifiable and analytical tool to unravel the relationships among cellular entities that govern the cellular functions [22].

All-against-all metabolite and gene expression profile comparisons for Adp, AdpGal, and Stat matrices were used to generate evolution-specific co-expression networks constructed using *r *(PCC). There was a significant, strong dependence between co-expression and functional relevance of the networks, attesting to the potential of co-expression network analysis (Figure 6a). In co-expression networks, nodes correspond to genes or metabolites, and edges link two genes or metabolites if they have a threshold correlation coefficient (*r*) at or above which genes or metabolites are considered to be changed differentially, exhibiting similar behavior. Correlation networks as such inherently contain corresponding large noise components, which were largely eliminated by setting the threshold of *r *at 0.9. The correlation networks based on the high threshold *r *of 0.9 reported here are less likely to contain noise while being sufficiently dense for analyses of topologic properties.

### Evaluation of evolution-specific networks

With respect to a number of parameters describing their common topologic properties, all evolution-specific co-expression networks (Adp: 4,170 nodes and 23,086 edges; AdpGal: 4,136 nodes and 20,501 edges; and Stat: 4,166 nodes and 54,028 edges) were found to be similar except for the average degree (see Additional data file 8). The average degree (*<k>*) is the average number of edges per node [22]. The Stat co-expression network exhibits higher *<k> *than do the Adp and AdpGal networks, which is consistent with its greater numbers of edges. The parameter *<k> *gives only a rough approximation of how dense the network is. The average clustering coefficient (*<C>*) is a measure of network density and characterizes the overall tendency of nodes to form clusters [22]. For all of the evolution-specific coexpression networks, *<C> *was approximately constant and high (about 0.05) when compared with randomly generated networks of similar size, for which the observed *<C> *was quite low (about 0.0008). The average path length *<l> *is the average shortest path between all pairs of nodes [22]. For all of the evolution-specific co-expression networks, the <*l*> was approximately constant and low (about 6.97; Figure 6e). When analyzing the networks' generic features, the clustering coefficients *C*(*k*) of all of the networks were more or less constant, implying that they did not exhibit a hierarchical structure (Figure 6b). The node degree (*k*) distribution of all of the networks appeared to have an exponential drop-off in the tail, following a power law (Figure 6c). Overall, these evaluations suggest that the global properties of these evolution-specific co-expression networks are indistinguishable.

### Evolution-specific intersection networks

Strain-specific and evolution-specific networks were screened for the set of nodes *N*, for which there is a link (*r *≥ 0.9) between two nodes *a *and *b *in both strains in the particular evolution type, in order to build evolution-specific intersection networks. By examining the intersection networks of both strains, we found that the path length distribution varied among networks. All intersection networks differed in *<k>*, which is consistent with their varying numbers of edges. The average clustering coefficient *<C> *was slightly higher in the Adp intersection network (*<C> *Adp intersection = 0.113, AdpGal intersection = 0.07, and Stat intersection = 0.089), demonstrating high network density and tendency of nodes to form clusters in the Adp intersection network (see Additional data file 8). The average path length *<l> *was almost equal in all cases, but its distribution in the Adp intersection network differed, indicating high network navigability (Figure 6f, g). Based on the observations of the global properties of the evolution-specific intersection networks, the Adp intersection network can be distinguished from other intersection networks, demonstrating its unique characteristics.

### Parallelism and functional relevance of molecular evolution

The generated networks were examined for functional coherence by assigning GO functional annotations to the networks' entities, and the level of parallelism in the representation of these functional categories was elucidated. Parallel evolution is the independent development of similar traits in distinct but evolutionarily related lineages through similar selective factors on both lines [23]. Parallel evolution of similar traits across both lines are used as an indicator that the change is adaptive [24]. Previous studies in *E. coli *and *Saccharomyces cerevisiae *have demonstrated parallel changes in independently adapted lines of replicate populations by utilizing gene expression profiling [17,19]. Here, we examined the parallelism of metabolite and gene expression levels among the evolved lines of different populations that exhibited similar growth behavior.

**Figure 7:**
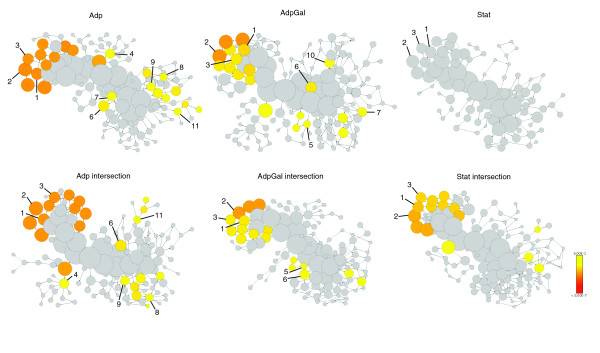
Parallelism and functional relevance of molecular evolution. Gene Ontology (GO) functional annotations were mapped to the corresponding evolution-specific co-expression networks and examined for commonalities in the co-expressed entities representing functional related categories. Each node represents a GO functional category, and the area of a node is proportional to the number of genes in the network matrix to the corresponding GO category. Statistically and significantly over-represented categories are color coded based on the hypergeometric test *P *value, which was corrected by Benjamini & Hochberg false discovery rate (a false discovery rate-controlled *P *value cutoff of ≤ 0.05). Gray nodes are not significantly over-represented. **(a-c) **GO annotations were mapped to the evolution-specific co-expression networks, namely Adp (panel a), AdpGal (panel b), and Stat (panel c). **(d-f) **GO annotations mapped evolution-specific intersection co-expression networks, namely **(d) **Adp intersection, **(e) **AdpGal intersection, and **(f) **Stat intersection. Not all over-represented categories are labeled because of the interdependency of functional categories in the GO hierarchy. Definitions of numbers: 1, membrane; 2, cell wall (*sensu *bacteria); 3, inner membrane; 4, transporter activity; 5, transport; 6, catabolism; 7, cellular catabolism; 8, amino acid metabolism; 9, nitrogen compound metabolism; 10, carbohydrate metabolism; 11, energy derivation by oxidation of organic compounds.

To examine the functional coherence and parallelism among the evolutionary processes, we mapped the GO functional annotations to the corresponding evolution-specific co-expression networks and we attempted to address the extent to which these co-expressed entities represent functionally related categories. By mapping GO functional categories to the co-expression networks, statistically and significantly over-represented functional categories were color coded according to the hypergeometric test *P *value, which was corrected by Benjamini & Hochberg false discovery rate (a false discovery rate-controlled *P *value cutoff of ≤ 0.05; Figure 7a-f). To examine the parallelism of evolutionary processes in both of the strains within the context of GO functional categories, we mapped the GO functional annotations to the co-expression networks (*r *≥ 0.9) generated by merging the data matrix of both strains, forming three evolution-specific co-expression networks, namely Adp, AdpGal, and Stat networks (Figure 7a, b, c). The level of parallelism differed among these networks. In the Adp network, for example, membrane, cell wall (*sensu *bacteria), inner membrane, transport activity, catabolism, and cellular catabolism functional categories were significantly over-represented (*P *≤ 0.05; Figure 7a). In the AdpGal network, membrane, cell wall (*sensu *bacteria), inner membrane, transport, catabolism, and cellular catabolism functional categories were over-represented (*P *≤ 0.05; Figure 7b). However, in the Stat network, none of the GO functional categories was significantly over-represented, denoting decreased level of parallelism among both strains (Figure 7c). Further examination of parallelism of evolutionary processes was extended to intersection co-expression networks (Figure 7d, e, f), which were created by selecting the nodes that are connected (*r *≥ 0.9) in both the strains in the particular evolutionary process in question. By examining the parallelism in these intersection co-expression networks, apart from other functional categories, we found that the commonly observed distribution of statistically over-represented GO categories in all of the co-expression networks belonged to membrane-associated GO functional categories (Figure 7d, e, f).

### Parallelism in outer-membrane protein expression

**Figure 8:**
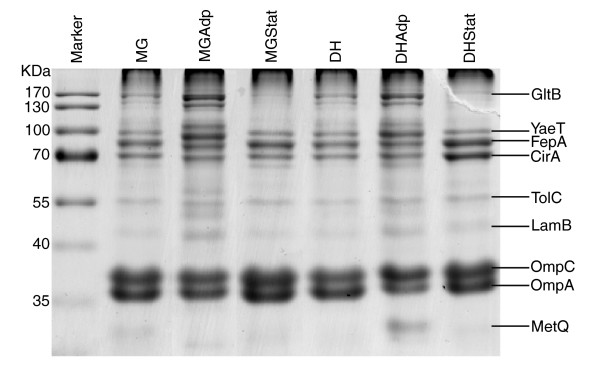
Parallelism and functional significance in the outer membrane protein expression. SDS gel electrophoresis of the protein samples obtained from the outer membrane of the ancestor and evolved lines showing the identified proteins by peptide mass fingerprinting.

To further examine the extent of parallel evolutionary changes, we determined the expression levels of proteins associated with the outer membrane (OM) of the ancestor and evolved strains, whose membrane-related GO functional categories were over-represented in the evolution-specific co-expression networks (Figure 7a-f). OM protein levels revealed substantial differential expression among the ancestor and evolved strains (Figure 8). In Adp lines, GltB (glutamate synthase [nicotinamide adenine dinucleotide phosphate (NADPH)] large chain precursor), LamB (maltose high-affinity receptor), and YaeT (polypeptide involved in outer-membrane protein biogenesis) proteins were over-expressed; whereas in Stat lines FepA (outer receptor for ferric enterobactin), CirA (outer membrane receptor for iron-regulated colicin I receptor), OmpC (outer membrane porin), and OmpA (outer-membrane porin) proteins were differentially over-expressed (Figure 8). Significantly, we observed parallelism in the level of protein expression patterns in these evolved strains and involvement of the outer membrane proteins in these evolutionary processes.

## Discussion

In this study we examined the metabolome and transcriptome profiles of excess nutrient adaptive evolution, pleiotropic environmental shift changes, and prolonged stationary phase evolution in two strains of *E. coli *K-12. We found significant influence of genes involved in transport and membrane related functional categories in all evolutionary conditions evaluated in this study. In earlier studies, during prolonged nutrient limited chemostat culture of bacterial populations, it was reported that the populations tend toward mutational adaptation in transport systems in order to increase the efficiency with which they utilize limited nutrients [25-28]. For example, glucose limited chemostat evolved strains attained diverse mutations at several loci in LamB porin, which increased glucose permeability [27-29]. An earlier study of adaptation of *Ralstonia *in selective environments [30] resulted in morphologic changes in the outer cell envelope in all of the lineages examined.

**Figure 9:**
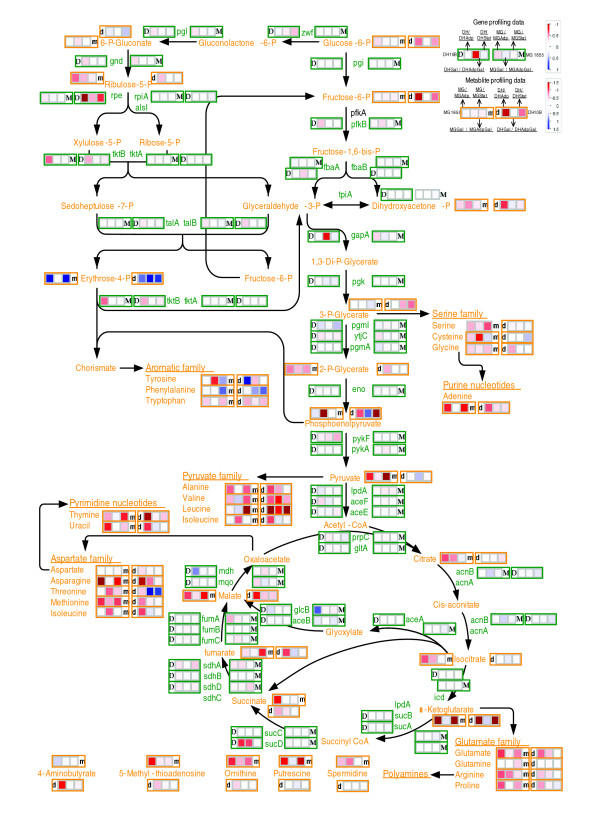
Gene and metabolite levels in the central metabolic routes and the diversion of key intermediates to biosynthetic pathways. Genes are represented in green text, and metabolites in orange text. Ancestor and evolved strain-specific gene expression comparisons are denoted in green boxes (M, MG1655; D, DH10B). Ancestor and evolved strain-specific metabolite abundance comparisons are denoted in orange boxes (m, MG1655; d, DH10B). Logarithmically transformed (to base 2) response ratios were utilized for each comparison according to the log_2 _ratio scale on the upper right inset.

In adaptation to excess nutrient resources, the Adp lines exhibited higher levels of metabolites that are involved in the nucleotide pathway and TCA cycle and its intermediates (Figures 1, 3, and 8). In line with these observations, the expression levels of genes involved in these pathways were also over-expressed in the Adp lines (Figure 9; also see Additional data file 5). Specifically, the pentose phosphate pathway (produces pentose phosphates for nucleic acid synthesis) was differentially regulated, along with the histidine biosynthesis pathway, which shares metabolites with the purine and nucleotide biosynthesis pathways (see Additional data files 6 and 9). For example, glutamate, which is involved in the *de novo *biosynthesis of purine nucleotides and various other pathways as a reactant, was accumulated in higher amounts in the Adp lines. In accordance with this observation, the genes that are involved in the glutamate biosynthesis and the protein glutamate synthase (GltB) were upregulated in the Adp lines (Figure 8). Taken together, the increased growth fitness in Adp lines, relative to their ancestor lines, can be presumed to be due to the differential levels of TCA cycle components (the first step in generating precursors for several biosynthetic pathways) and components involved in pentose phosphate pathway (the main source of precursor metabolites for biosynthesis and the main producer of NADPH, which is utilized in several biosynthesis pathways). However, the involvement of these pathways in growth fitness requires confirmation in additional studies. Our finding that central metabolism is altered in excess nutrient and famine conditions (Figure 9) is consistent with a previously reported study focusing on adaptive evolution in yeast in glucose-limited chemostat experiments, which demonstrated gene expression variation in glycolysis, the TCA cycle, and metabolite transport [17].

In long-term stationary phase cultures, cells lose their integrity and release their cellular components into the medium as cells enter the death phase [2]. For cell maintenance and growth, the surviving cells scavenge nutrient sources from the cellular debris (amino acids from proteins, carbohydrates from the cell wall, and lipids from cell membrane material and DNA) of their dead siblings [2]. This nutrient scavenging process due to nutrient limitation enhances the availability of carbon sources by reconstruction of the OM composition (glycerophospholipids, lipopolysaccharides and proteins) and there by improving the permeability of the OM [31]. The OM of *E. coli *consists of a lipid bilayer structure composed of an outer layer consisting of lipopolysaccharide and an inner layer consisting of phospholipids [32]. The genes involved in the biosynthetic pathways of fatty acids (key building blocks for the phospholipid components of cell membranes) and lipids were over-expressed in Stat lines (see Additional data file 10). Other major components of the OM are proteins; these largely consist of porins, which co-exist with lipopolysaccharide [33]. The OM of the cell is the first point of contact with the external environment, and therefore its cellular constituents may be the most sensitive to the external environment. Consistent with this hypothesis, OM proteins FepA, CirA, OmpC, and OmpA were differentially over-expressed in Stat lines (Figure 8), and the genes belonging to the membrane-associated GO functional categories were significantly over-represented in the corresponding evolutionary networks as well (Figure 7f). This demonstrates the reliability of the correlation network analysis, which was sufficiently robust to identify significant changes in the integrated metabolite and gene profiling dataset.

Mutation rates in stationary phase are known to be influenced by the genetic background of the strain [10]. Initial isogenic long-term stationary phase cultures are highly dynamic and are known to yield different 'growth advantage in stationary phase' mutations due to significant genotypic diversity in these cultures [2]. Consistent with this hypothesis, when we applied PCA (Figure 5c, f) and correlation plot analysis (Figure 5i, l), the metabolite and gene expression levels of Stat lines exhibited low degrees of parallelism when compared with their ancestor lines. Likewise, when GO functional annotations were mapped onto the Stat co-expression network, we found that none of the GO functional categories was significantly over-represented, denoting a low level of parallelism (Figure 7c). However, when applied to the Stat intersection co-expression network, membrane-associated GO functional categories were significantly over-represented (Figure 7f). These observations demonstrate the parallelism in membrane-associated categories in the Stat intersection co-expression network but not in the Stat co-expression network. It suggests the existence of parallelism in membrane-associated categories but not in similar membrane-associated genes in Stat lines. From this we can conclude that distinct but functionally related genes are involved in the parallelism in the Stat intersection co-expression network.

## Conclusion

We analyzed two different strains under three different evolutionary conditions. Integration of metabolome and gene expression data within the context of evolution facilitated investigation of the path of evolution and their degree of parallelism. Classifying microarray data according to significantly over-represented GO functional categories showed that the transport related categories had the greater overall representation. Similarly, by mapping the GO annotation to the correlation networks, we found that the membrane associated functional categories were significantly over-represented. The OM of the cell is the first point of contact with the external environment, which acts as a barrier that is quite resistant to insult and acts as a channel for nutrient transport. Components of the OM may therefore be the cellular constituents that are most sensitive to the external environment. Analyses of the OM proteins of the ancestor and evolved strains revealed clear differential regulation of the OM proteins.

In summary, all of the evolutionary experiments reported in this study demonstrate the vital role played by the involvement of the membrane associated components in the evolutionary process. These studies show that adaptive evolution in excess nutrient conditions are appropriate for examining the extent of parallelism in the evolutionary process of the evolved populations, whereas the prolonged stationary phase conditions are useful in understanding the evolution of microbial diversity among evolved populations and the dynamic state of the evolved condition. Such studies will certainly advance our understanding of the process of evolution immensely and, along with constructed models [34], will be an ideal initial source of data for systems biology study of microbial evolution.

## Materials and methods

### Strain and culture conditions

Both the bacterial strains MG1655 and DH10B used in this study are derivatives of *E. coli *K-12. All of the experiments were conducted in 250 ml of M9 minimal medium supplemented with 4 g/l glucose or galactose in covered 1 l Erlenmeyer flasks at 37°C. Adaptation to excess nutrient experiments were carried out in the presence of 4 g/l glucose through serial passage at exponential phase for about 1,000 generations. The cells were grown overnight and were diluted by passage into fresh medium. Passage of each culture into fresh medium was conducted in a laminar flow station using standard sterile technique practices. Serial passage was conducted for 37 days at exponential phase for about 1,000 generations. For adaptation due to environmental shift experiments, the strains that were adapted to excess nutrient (glucose) condition for about 1,000 generations were grown in 4 g/l galactose. For prolonged stationary phase adaptation experiments, both the strains were incubated for 37 days in M9 minimal medium with 4 g/l glucose as initial source of carbon. The evolved populations were frozen using liquid nitrogen and stored in a freezer at -80°C.

### Metabolite profiling

Approximately equal numbers of cells (7 × 10^9^) were taken from the exponential phase of growth for all of the experiments. Cells were disrupted using acid washed glass beads at maximum speed in a Ribolyser (Q-BIOgene, Heidelberg, Germany) at a setting of 6.5 m/second, twice for 45 seconds in the presence of 80% methanol. Subsequently, metabolites were derived using methoxylamine hydrochloride and *N*-methyl-*N*-(trimethylsilyl)trifluoroacetamide in the presence of ribitol as the internal standard. Sample volumes of 1 μl were analysed using a TraceGC gas chromatograph coupled to a PolarisQ ion trap mass spectrometer (Thermo Finnigan, Dreieich, Germany). Derived metabolites were evaporated at 250°C in splitless mode and separated on a 30 m × 0.25 mm Equity-5 column with 0.25 μm coating (Supelco, Bellefonte, California, USA). Metabolites were identified by comparison with purified standards, the NIST 2005 database (NIST) and the Golm Metabolome Database [35]. Selected metabolite peak areas were automatically quantified using the processing setup implemented in the Xcalibur 1.4 software (Thermo Finnigan, Dreieich, Germany). The relative response ratios calculated from the peak areas were normalized by the internal standard ribitol and dry mass of the sample. For both the strains in all the biologic experiments, six replicates were used, which consisted of three independent biologic replicates and three technical replicates. The variation among the biological replicates was estimated to be relatively low (see Additional data file 11 [part a]).

### Gene expression profiling

*E. coli K12 V2 OciChip*™ arrays containing 4,288 gene specific oligonucleotide probes representing the complete *E. coli *K-12 genome were utilized in this study (Ocimum Biosolutions, Hyderabad, India). Total RNA was isolated using RNeasy kit (Qiagen, Hilden, Germany), in accordance with the manufacturer's instructions. Reverse transcription, labeling, and scanning were performed as described previously [36]. Hybridization was carried out in accordance with the manufacturer's instructions (Ocimum Biosolutions, Hyderabad, India).

### Microarray data analysis

Mean signal and mean local background intensities were determined for each spot of the microarray images, by using the ImaGene 6.0 software for spot detection, image segmentation, and signal quantification (Biodiscovery, Los Angeles, California, USA). After subtraction of the local background intensities from the signal intensities, the average intensity in both channels was subsequently normalized using the LOWESS (locally weighted scatterplot smoothing) method using the GeneSight 4.0 software package (Biodiscovery, Los Angeles, California, USA). The normalized log_2 _ratios were used to represent the data graphically and to calculate Wilcoxon rank sum test *P *value using MapMan software [37], with functional classifications based on MultiFun and GO terms, a cell function assignment scheme, with slight modification [38,39]. The SAM add-in to Microsoft Excel was used for comparisons of replicate array experiments [16]. For both of the strains in all of the biologic experiments, three or more replicates were used, which consisted of three biologic replicates. The variation among the biologic replicates was estimated to be relatively low (see Additional data file 11 [part b]). The ArrayExpress repository [40] accession number for the microarray data is E-MEXP-1166, which consists of 29 hybridizations.

### Network analysis

All of the networks reported in this study were constructed based on PCC *r *≥ 0.9 measure (nodes that correspond to genes or metabolites with *r *≥ 0.9 were linked by an edge). All-against-all metabolite and gene expression profile *r *values of evolution-specific matrices were used to generate evolution-specific co-expression network. Strain-specific and evolution-specific matrices were used to generate evolution-specific intersection co-expression network. Intersection co-expression networks are the network over the set of nodes *N*, where there is a link (*r *≥ 0.9) between two nodes *i *and *j *if they are connected in both of the strains in the particular evolutionary condition in context. Topologic properties of the networks were analyzed using the Pajek program [41].

### Network functional analysis

Network visualization and functional analysis was achieved using Cytoscape [42]. Networks were screened for highly linked clusters of genes or metabolites using MCODE [43]. Genes in the networks were functionally categorized using their GO biologic process annotation terms [44], and the over-represented GO terms were identified with BINGO [45]. The hypergeometric test was used for this purpose, with the Benjamini and Hochberg false discovery rate correction (a false discovery rate-controlled *P *value cutoff of ≤ 0.05).

### Outer membrane protein analysis

Approximately equal numbers of extracted cells (7 × 10^9^) were disrupted by ultrasonication with 5 ml of 50 mmol/l Tris/HCl (pH 7.3), containing 0.7 mg of DNase I (Sigma, Taufkirchen, Germany) and 0.5 mmol/l protease inhibitor (Pefabloc SC; Centerchem, Inc., Norwalk, CT, USA). After the unbroken cells were removed by centrifugation, the supernatant was treated with ice-cold 0.1 mol/l sodium carbonate (pH 11). Eventually, the carbonate treated membranes were collected and subsequently analysed by SDS one-dimensional gel electrophoresis. Excised protein bands were subjected to tryptic digestion and mass spectra were obtained on a Ultraflex MALDI-TOF/TOF (Bruker Daltonics, Bremen, Germany). Peptide masses were searched against the *E. coli *database located on our local server using MASCOT search engine (Matrix Science Ltd., London, U.K) with a mass cutoff of 100 ppm.

## Abbreviations

*<C>*, clustering coefficient; GO, Gene Ontology; *<k>*, average degree; <*l*>, average path length; NADPH, nicotinamide adenine dinucleotide phosphate; OM, outer membrane; PCA, principal components analysis; PCC, Pearson correlation coefficient; SAM, significance analysis of microarrays; TCA, tricarboxylic acid.

## Authors' contributions

CV conducted all the experiments cited in this study, analyzed the results, and wrote this manuscript. A Barsch was involved in metabolomics experiments. KF was involved in experimental guidance. KN was involved in experimental design. A Becker is the scientist in whose laboratory microarray experiments were conducted. EF is the scientist in whose laboratory all of the experiments were conducted and was involved in the experimental design.

## Additional data files

The following additional data are available with the online version of this paper. Additional data file 1 is a table listing the identified metabolites of the ancestral and evolved strains by gas chromatography-mass spectrometry. Additional data file 2 is a table listing significantly altered metabolites in all of the evolved conditions. Additional data file 3 is a table listing significantly altered genes in all of the evolved conditions. Additional data file 4 is a table listing significant GO functional categories involved in all of the evolved conditions. Additional data file 5 is a figure showing the integration of transcriptome and metabolome data during the comparison of ancestral and evolved strains in excess nutrient adaptive evolution. Additional data file 6 is a figure showing the gene expression and metabolite abundance level in the pentose phosphate pathway in excess nutrient adapted strains. Additional data file 7 is a figure showing PCA analyses for both the ancestor and evolved lines of both the strains grown in two different media. Additional data file 8 is a table listing common topologic properties of all evolution co-expression networks. Additional data file 9 is a figure showing the gene expression and metabolite abundance level in histidine biosynthesis pathway in excess nutrient adapted strains. Additional data file 10 is a figure showing the integration of transcriptome and metabolome data during the comparison of ancestral and evolved strains in prolonged stationary phase evolution. Additional data file 11 is a figure showing metabolite abundance level and gene expression level among the biologic replicates.

## Supplementary Material

Additional data file 1 Presented is a table listing the identified metabolites of the ancestral and evolved strains by gas chromatography-mass spectrometry. Click here for file

Additional data file 2 Presented is a table listing significantly altered metabolites in all of the evolved conditions. Click here for file

Additional data file 3 Presented is a table listing significantly altered genes in all of the evolved conditions. Click here for file

Additional data file 4 Presented is a table listing significant GO functional categories involved in all of the evolved conditions. Click here for file

Additional data file 5 Presented is a figure showing the integration of transcriptome and metabolome data during the comparison of ancestral and evolved strains in excess nutrient adaptive evolution. Click here for file

Additional data file 6 Presented is a figure showing the gene expression and metabolite abundance level in the pentose phosphate pathway in excess nutrient adapted strains. Click here for file

Additional data file 7 Presented is a figure showing PCA analyses for both the ancestor and evolved lines of both strains grown in two different media. Click here for file

Additional data file 8 Presented is a table listing common topologic properties of all evolution co-expression networks. Click here for file

Additional data file 9 Presented is a figure showing the gene expression and metabolite abundance level in histidine biosynthesis pathway in excess nutrient adapted strains. Click here for file

Additional data file 10 Presented is a figure showing the integration of transcriptome and metabolome data during the comparison of ancestral and evolved strains in prolonged stationary phase evolution. Click here for file

Additional data file 11 Presented is a figure showing metabolite abundance level and gene expression level among the biologic replicates. Click here for file

## References

[B1] Elena SF Lenski RE Evolution experiments with microorganisms: the dynamics and genetic bases of adaptation. Nat Rev Genet 2003 4 457 469 10.1038/nrg1088 12776215

[B2] Finkel SE Long-term survival during stationary phase: evolution and the GASP phenotype. Nat Rev Microbiol 2006 4 113 120 10.1038/nrmicro1340 16415927

[B3] Wright BE Stress-directed adaptive mutations and evolution. Mol Microbiol 2004 52 643 650 10.1111/j.1365-2958.2004.04012.x 15101972

[B4] Zinser ER Kolter R *Escherichia coli *evolution during stationary phase. Res Microbiol 2004 155 328 336 10.1016/j.resmic.2004.01.014 15207864

[B5] Lenski RE Travisano M Dynamics of adaptation and diversification: a 10,000-generation experiment with bacterial populations. Proc Natl Acad Sci USA 1994 91 6808 6814 10.1073/pnas.91.15.6808 8041701 PMC44287

[B6] Sniegowski PD Gerrish PJ Lenski RE Evolution of high mutation rates in experimental populations of *E. coli*. Nature 1997 387 703 705 10.1038/42701 9192894

[B7] Papadopoulos D Schneider D Meier-Eiss J Arber W Lenski RE Blot M Genomic evolution during a 10,000-generation experiment with bacteria. Proc Natl Acad Sci USA 1999 96 3807 3812 10.1073/pnas.96.7.3807 10097119 PMC22376

[B8] Finkel SE Kolter R Evolution of microbial diversity during prolonged starvation. Proc Natl Acad Sci USA 1999 96 4023 4027 10.1073/pnas.96.7.4023 10097156 PMC22413

[B9] Loewe L Textor V Scherer S High deleterious genomic mutation rate in stationary phase of *Escherichia coli*. Science 2003 302 1558 1560 10.1126/science.1087911 14645846

[B10] Bjedov I Tenaillon O Gerard B Souza V Denamur E Radman M Taddei F Matic I Stress-induced mutagenesis in bacteria. Science 2003 300 1404 1409 10.1126/science.1082240 12775833

[B11] Lombardo MJ Aponyi I Rosenberg SM General stress response regulator RpoS in adaptive mutation and amplification in *Escherichia coli*. Genetics 2004 166 669 680 10.1534/genetics.166.2.669 15020458 PMC1470735

[B12] Zinser ER Kolter R Prolonged stationary-phase incubation selects for lrp mutations in *Escherichia coli *K-12. J Bacteriol 2000 182 4361 4365 10.1128/JB.182.15.4361-4365.2000 10894750 PMC101964

[B13] Zinser ER Kolter R Mutations enhancing amino acid catabolism confer a growth advantage in stationary phase. J Bacteriol 1999 181 5800 5807 10482523 10.1128/jb.181.18.5800-5807.1999 PMC94102

[B14] Raamsdonk LM Teusink B Broadhurst D Zhang N Hayes A Walsh MC Berden JA Brindle KM Kell DB Rowland JJ Westerhoff HV van Dam K Oliver SG A functional genomics strategy that uses metabolome data to reveal the phenotype of silent mutations. Nat Biotechnol 2001 19 45 50 10.1038/83496 11135551

[B15] Fernie AR Trethewey RN Krotzky AJ Willmitzer L Metabolite profiling: from diagnostics to systems biology. Nat Rev Mol Cell Biol 2004 5 763 769 10.1038/nrm1451 15340383

[B16] Tusher VG Tibshirani R Chu G Significance analysis of microarrays applied to the ionizing radiation response. Proc Natl Acad Sci USA 2001 98 5116 5121 10.1073/pnas.091062498 11309499 PMC33173

[B17] Ferea TL Botstein D Brown PO Rosenzweig RF Systematic changes in gene expression patterns following adaptive evolution in yeast. Proc Natl Acad Sci USA 1999 96 9721 9726 10.1073/pnas.96.17.9721 10449761 PMC22277

[B18] Riehle MM Bennett AF Lenski RE Long AD Evolutionary changes in heat-inducible gene expression in lines of *Escherichia coli* adapted to high temperature. Physiol Genomics 2003 14 47 58 12672900 10.1152/physiolgenomics.00034.2002

[B19] Cooper TF Rozen DE Lenski RE Parallel changes in gene expression after 20,000 generations of evolution in *Escherichia coli*. Proc Natl Acad Sci USA 2003 100 1072 1077 10.1073/pnas.0334340100 12538876 PMC298728

[B20] Fong SS Joyce AR Palsson BO Parallel adaptive evolution cultures of *Escherichia coli *lead to convergent growth phenotypes with different gene expression states. Genome Res 2005 15 1365 1372 10.1101/gr.3832305 16204189 PMC1240078

[B21] Wolfe CJ Kohane IS Butte AJ Systematic survey reveals general applicability of 'guilt-by-association' within gene coexpression networks. BMC Bioinformatics 2005 6 227 10.1186/1471-2105-6-227 16162296 PMC1239911

[B22] Barabasi AL Oltvai ZN Network biology: understanding the cell's functional organization. Nat Rev Genet 2004 5 101 113 10.1038/nrg1272 14735121

[B23] Schluter D Parallel evolution and inheritance of quantitative traits. Am Nat 2004 163 809 822 10.1086/383621 15266380

[B24] Bull JJ Badgett MR Wichman HA Huelsenbeck JP Hillis DM Gulati A Ho C Molineux IJ Exceptional convergent evolution in a virus. Genetics 1997 147 1497 1507 9409816 10.1093/genetics/147.4.1497 PMC1208326

[B25] Helling RB Vargas CN Adams J Evolution of *Escherichia coli *during growth in a constant environment. Genetics 1987 116 349 358 3301527 10.1093/genetics/116.3.349 PMC1203146

[B26] Sonti RV Roth JR Role of gene duplications in the adaptation of *Salmonella typhimurium *to growth on limiting carbon sources. Genetics 1989 123 19 28 2680755 10.1093/genetics/123.1.19 PMC1203782

[B27] Notley-McRobb L Ferenci T Adaptive mgl-regulatory mutations and genetic diversity evolving in glucose-limited *Escherichia coli *populations. Environ Microbiol 1999 1 33 43 10.1046/j.1462-2920.1999.00002.x 11207716

[B28] Notley-McRobb L Ferenci T The generation of multiple co-existing mal-regulatory mutations through polygenic evolution in glucose-limited populations of *Escherichia coli*. Environ Microbiol 1999 1 45 52 10.1046/j.1462-2920.1999.00003.x 11207717

[B29] Notley-McRobb L Ferenci T Experimental analysis of molecular events during mutational periodic selections in bacterial evolution. Genetics 2000 156 1493 1501 11102352 10.1093/genetics/156.4.1493 PMC1461358

[B30] Riley MS Cooper VS Lenski RE Forney LJ Marsh TL Rapid phenotypic change and diversification of a soil bacterium during 1000 generations of experimental evolution. Microbiology 2001 147 995 1006 11283295 10.1099/00221287-147-4-995

[B31] Liu X Ferenci T An analysis of multifactorial influences on the transcriptional control of ompF and ompC porin expression under nutrient limitation. Microbiology 2001 147 2981 2989 11700349 10.1099/00221287-147-11-2981

[B32] Nikaido H Nakae T The outer membrane of Gram-negative bacteria. Adv Microb Physiol 1979 20 163 250 394591 10.1016/s0065-2911(08)60208-8

[B33] Nikaido H Molecular basis of bacterial outer membrane permeability revisited. Microbiol Mol Biol Rev 2003 67 593 656 10.1128/MMBR.67.4.593-656.2003 14665678 PMC309051

[B34] Covert MW Knight EM Reed JL Herrgard MJ Palsson BO Integrating high-throughput and computational data elucidates bacterial networks. Nature 2004 429 92 96 10.1038/nature02456 15129285

[B35] Kopka J Schauer N Krueger S Birkemeyer C Usadel B Bergmuller E Dormann P Weckwerth W Gibon Y Stitt M Willmitzer L Fernie AR Steinhauser D GMD@CSB.DB: the Golm Metabolome Database. Bioinformatics 2005 21 1635 1638 10.1093/bioinformatics/bti236 15613389

[B36] Ruberg S Tian ZX Krol E Linke B Meyer F Wang Y Puhler A Weidner S Becker A Construction and validation of a *Sinorhizobium meliloti *whole genome DNA microarray: genome-wide profiling of osmoadaptive gene expression. J Biotechnol 2003 106 255 268 10.1016/j.jbiotec.2003.08.005 14651866

[B37] Thimm O Blasing O Gibon Y Nagel A Meyer S Kruger P Selbig J Muller LA Rhee SY Stitt M MAPMAN: a user-driven tool to display genomics data sets onto diagrams of metabolic pathways and other biological processes. Plant J 2004 37 914 939 10.1111/j.1365-313X.2004.02016.x 14996223

[B38] Serres MH Riley M MultiFun, a multifunctional classification scheme for *Escherichia coli* K-12 gene products. Microb Comp Genomics 2000 5 205 222 11471834 10.1089/omi.1.2000.5.205

[B39] Serres MH Goswami S Riley M GenProtEC: an updated and improved analysis of functions of *Escherichia coli *K-12 proteins. Nucleic Acids Res 2004 32 Database D300 D302 10.1093/nar/gkh087 14681418 PMC308821

[B40] Parkinson H Kapushesky M Shojatalab M Abeygunawardena N Coulson R Farne A Holloway E Kolesnykov N Lilja P Lukk M Mani R Rayner T Sharma A William E Sarkans U Brazma A ArrayExpress: a public database of microarray experiments and gene expression profiles. Nucleic Acids Res 2007 35 Database D747 D750 10.1093/nar/gkl995 17132828 PMC1716725

[B41] Batagelj V Mrvar A PAJEK: program for large network analysis. Connections 1998 21 47 57

[B42] Shannon P Markiel A Ozier O Baliga NS Wang JT Ramage D Amin N Schwikowski B Ideker T Cytoscape: a software environment for integrated models of biomolecular interaction networks. Genome Res 2003 13 2498 2504 10.1101/gr.1239303 14597658 PMC403769

[B43] Bader GD Hogue CW An automated method for finding molecular complexes in large protein interaction networks. BMC Bioinformatics 2003 4 2 10.1186/1471-2105-4-2 12525261 PMC149346

[B44] Ashburner M Ball CA Blake JA Botstein D Butler H Cherry JM Davis AP Dolinski K Dwight SS Eppig JT Harris MA Hill DP Issel-Tarver L Kasarskis A Lewis S Matese JC Richardson JE Ringwald M Rubin GM Sherlock G Gene ontology: tool for the unification of biology. The Gene Ontology Consortium. Nat Genet 2000 25 25 29 10.1038/75556 10802651 PMC3037419

[B45] Maere S Heymans K Kuiper M BiNGO: a Cytoscape plugin to assess overrepresentation of gene ontology categories in biological networks. Bioinformatics 2005 21 3448 3449 10.1093/bioinformatics/bti551 15972284

