# Supplementary material for: Perceiving molecular evolution processes in Escherichia coli by comprehensive metabolite and gene expression profiling
Source: Genome Biol. 2008 Apr 10;9(4):R72. doi: 10.1186/gb-2008-9-4-r72 (PMC2643943; doi:10.1186/gb-2008-9-4-r72)
Supplement: Additional data file 1 — Presented is a table listing the identified metabolites of the ancestral and evolved strains by gas chromatography-mass spectrometry. [file gb-2008-9-4-r72-S1.pdf]

Identified metabolites of the ancestral and evolved strains by gas chromatography-mass spectrometry. Response ratios of metabolites having *P*-Value ≤ 0.05 are shown in bold. Metabolites having correlation coefficient ≥ 0.5 are underlined. Response ratios of metabolites having *P*-Value ≤ 0.05 and correlation coefficient ≥ 0.5 are shown in bold underline.

| **Metabolites** | **MG / MG_Gal** | **MG / MG_Adp** | **MG / MG_AdpGal** | **MG / MG_stat** | **MG_Gal / MG_AdpGal** | **MG_Adp / MG_AdpGal** | **MG_Adp / MG_stat** | **DH / DH_Gal** | **DH / DH_Adp** | **DH / DH_AdpGal** | **DH / DH_stat** | **DH_Gal / DH_AdpGal** | **DH_Adp / DH_AdpGal** | **DH_Adp / DH_stat** |
|---|---|---|---|---|---|---|---|---|---|---|---|---|---|---|
| 2-Aminoadipate (260) | 1.26 | 1.08 | 0.50 | 1.03 | 0.40 | 0.46 | 0.96 | 0.78 | 1.56 | 0.99 | 0.59 | 1.27 | 0.64 | 0.38 |
| 2-Hydroxyglutarate (203,247) | 1.38 | 0.69 | 0.91 | 1.24 | **0.66** | 1.33 | 1.81 | 1.34 | **1.84** | 1.41 | 1.53 | 1.05 | 0.77 | 0.83 |
| 2-Isopropylmalate (275) | 0.99 | 0.63 | 0.59 | 1.33 | 0.60 | 0.94 | 2.12 | 0.95 | 0.75 | **0.44** | 0.75 | **0.47** | 0.59 | 1.00 |
| 2-Ketoisocaproate (216) | 1.00 | 0.45 | 0.52 | 0.32 | 0.52 | 1.16 | 0.71 | 0.95 | 0.87 | 0.55 | 0.67 | 0.59 | 0.64 | 0.77 |
| 2-Methylcitrate (287) | 0.52 | 0.54 | 0.53 | 0.99 | 1.03 | 0.99 | 1.85 | 0.35 | 0.72 | **0.21** | 0.78 | 0.59 | **0.28** | 1.08 |
| 2-Methylisocitrate (259) | 3.02 | 0.75 | 3.02 | 1.21 | 1.00 | **4.04** | 1.62 | 2.64 | 0.69 | 1.25 | 0.53 | 0.47 | 1.80 | 0.77 |
| 4-Aminobutyrate (174) | 2.29 | 1.44 | 2.60 | 0.96 | 1.14 | **1.81** | 0.66 | **2.62** | 0.44 | **2.94** | 0.83 | 1.12 | **6.61** | 1.87 |
| 5-Methyl-thioadenosine (236) | 0.72 | **0.43** | **0.57** | 0.78 | 0.79 | 1.33 | 1.82 | 1.36 | **0.68** | 1.16 | 1.13 | 0.85 | **1.69** | **1.66** |
| Adenine (264) | 0.71 | **0.47** | 0.66 | 0.41 | 0.92 | 1.40 | 0.88 | 1.22 | **0.53** | 0.92 | 0.76 | 0.75 | **1.75** | 1.44 |
| Adenosine (236) | 1.10 | 1.05 | 1.50 | 2.09 | 1.36 | 1.42 | 1.99 | 0.84 | 1.35 | 2.70 | 1.06 | 3.20 | 1.99 | 0.78 |
| a-Glycerophosphate (357) | 0.97 | 0.66 | 0.85 | 0.55 | 0.88 | 1.30 | 0.84 | 0.99 | **0.67** | 0.99 | 0.91 | 1.00 | **1.49** | **1.36** |
| a-Ketoglutarate (198) | 0.54 | 0.20 | 0.73 | 0.15 | 1.34 | 3.65 | 0.78 | 1.41 | **0.19** | 2.03 | **0.26** | 1.45 | **10.94** | 1.41 |
| Alanine (116) | 0.67 | 0.55 | 0.73 | 0.54 | 1.08 | 1.33 | 1.00 | 1.58 | **0.51** | 1.01 | 0.89 | 0.64 | **1.96** | **1.73** |
| Arabinose (217) | 1.42 | 1.04 | 0.83 | **1.57** | **0.59** | 0.80 | 1.51 | 1.13 | 1.74 | 1.56 | 1.37 | 1.38 | 0.90 | 0.79 |
| Arginine (256) | 1.58 | 0.52 | 0.92 | 0.76 | 0.58 | 1.75 | 1.45 | **1.58** | 0.69 | 1.21 | 1.10 | 0.77 | 1.76 | 1.59 |
| Asparagine (216) | 0.65 | **0.33** | 0.53 | **0.41** | 0.83 | 1.62 | 1.25 | 1.16 | **0.37** | 0.66 | 0.81 | 0.57 | 1.79 | 2.19 |
| b-Alanine (248) | 1.80 | 0.81 | 1.13 | 1.39 | 0.63 | 1.39 | 1.71 | 1.01 | 1.08 | 1.23 | 1.23 | 1.21 | 1.13 | 1.13 |
| Citrate (257) | 1.06 | 0.52 | **0.62** | 1.25 | **0.59** | 1.20 | **2.41** | 1.24 | 1.01 | 1.11 | 0.77 | 0.89 | 1.10 | 0.76 |
| Cystathionine (128) | 0.91 | **0.19** | 0.28 | 0.61 | 0.31 | 1.44 | **3.17** | 0.87 | 0.35 | 0.54 | 1.41 | 0.62 | 1.54 | 4.07 |
| DHAP (400) | 0.88 | 0.75 | 0.87 | 0.82 | 0.98 | 1.16 | 1.09 | 1.70 | 0.51 | 1.98 | 0.83 | 1.16 | 3.91 | 1.65 |
| Diaminopimelate (200,272) | 1.33 | 0.57 | 1.03 | 0.47 | 0.77 | 1.81 | 0.83 | 1.13 | 1.01 | 1.62 | 1.50 | 1.43 | 1.60 | 1.48 |
| Erythrose-4-P (357) | 3.04 | 4.45 | 3.45 | 4.83 | 1.13 | 0.78 | 1.09 | 0.17 | 1.85 | 0.80 | 2.48 | 4.84 | 0.43 | 1.34 |
| Fructose-6-P (315) | 0.75 | 0.86 | 0.61 | 0.81 | 0.82 | 0.71 | 0.94 | 0.91 | **0.38** | 0.81 | **0.56** | 0.89 | **2.15** | 1.49 |
| Fructose (307) | **0.66** | 1.01 | **0.53** | 1.31 | 0.81 | **0.52** | 1.30 | **0.45** | 0.62 | **0.46** | 0.93 | 1.01 | 0.73 | 1.49 |

Identified metabolites of the ancestral and evolved strains by gas chromatography-mass spectrometry.

| | | | | | | | | | | | | | | |
|---|---|---|---|---|---|---|---|---|---|---|---|---|---|---|
| Fumarate (245) | 2.38 | 0.75 | 1.98 | 0.47 | 0.83 | 2.64 | 0.63 | 2.58 | 0.51 | 3.68 | 0.88 | 1.43 | 7.28 | 1.74 |
| Gluconate-6-P (387) | 1.20 | 1.02 | 0.91 | 1.06 | 0.76 | 0.89 | 1.03 | 0.89 | 1.28 | 0.90 | 1.51 | 1.01 | 0.71 | 1.18 |
| Gluconate (333) | 3.22 | 1.24 | 2.32 | 1.21 | 0.72 | 1.87 | 0.98 | 1.09 | 1.18 | 1.97 | 1.63 | 1.81 | 1.67 | 1.38 |
| Glucose (319) | 1.47 | 2.03 | 0.35 | 0.55 | 0.24 | 0.17 | 0.27 | 0.85 | 0.70 | 0.70 | 0.80 | 0.82 | 0.99 | 1.13 |
| Glucose-6-P (387) | 0.77 | 0.74 | 0.68 | 1.13 | 0.88 | 0.91 | 1.52 | 0.86 | 0.56 | 0.74 | 0.62 | 0.86 | 1.30 | 1.10 |
| Glucuronicacid (333) | 1.12 | 0.17 | 0.71 | 1.71 | 0.63 | 4.09 | 9.83 | 3.07 | 1.99 | 3.04 | 1.43 | 0.99 | 1.53 | 0.72 |
| Glutamate (230,246) | 0.90 | 0.50 | 0.71 | 0.58 | 0.79 | 1.42 | 1.17 | 1.70 | **0.55** | 1.18 | 0.95 | 0.69 | **2.12** | **1.71** |
| Glutamine (155) | 0.93 | 0.91 | 0.90 | 0.72 | 0.98 | 0.99 | 0.79 | 1.34 | 0.92 | 1.18 | 1.07 | 0.88 | 1.29 | 1.16 |
| Glycerate-2-P (299,315,459) | 1.00 | **0.58** | 0.68 | 0.62 | 0.68 | 1.16 | 1.06 | 1.12 | **0.65** | 1.13 | 0.99 | 1.02 | **1.74** | **1.52** |
| Glycerate (189,192) | 1.56 | 1.17 | 1.20 | 0.91 | 0.77 | 1.03 | 0.78 | 1.36 | 0.66 | 0.99 | 0.76 | 0.73 | 1.48 | 1.14 |
| Glycerate-3-P (227,299,459) | 1.29 | 1.04 | 1.35 | 1.33 | 1.05 | 1.30 | 1.28 | 1.17 | 0.98 | 0.76 | 0.56 | 0.65 | 0.77 | 0.57 |
| Glycine (174) | 1.30 | 0.72 | 1.00 | 0.68 | 0.77 | 1.39 | 0.95 | 0.94 | 0.63 | 1.10 | 0.95 | 1.17 | **1.74** | 1.50 |
| Isocitrate (245,319) | 1.00 | 0.53 | 0.64 | 1.15 | **0.64** | 1.20 | **2.18** | 0.86 | 1.04 | 1.10 | 0.89 | 1.29 | 1.07 | 0.86 |
| Isoleucine (158) | 2.12 | 0.90 | 1.15 | 0.92 | 0.54 | 1.28 | 1.02 | 1.02 | 0.51 | 1.36 | 0.87 | 1.33 | **2.67** | 1.70 |
| L,L-Cystathionine (218) | **0.38** | **0.39** | **0.37** | 0.65 | 0.96 | 0.93 | 1.66 | 0.59 | 0.92 | 0.46 | 0.66 | 0.78 | 0.50 | 0.71 |
| Lactate (191) | 1.03 | 0.80 | 0.66 | 0.54 | 0.64 | 0.83 | 0.67 | 1.01 | **0.61** | 0.89 | 0.93 | 0.88 | 1.46 | **1.52** |
| L-Aspartate (232) | 0.93 | 1.05 | 0.95 | 0.85 | 1.02 | 0.90 | 0.81 | 1.26 | 0.75 | 1.08 | 0.90 | 0.85 | 1.44 | 1.20 |
| L-Cysteine (220) | 1.28 | 0.71 | **0.54** | 1.40 | **0.42** | 0.76 | 1.99 | 0.97 | 1.02 | 0.95 | 1.54 | 0.97 | 0.93 | 1.50 |
| Leucine (158) | 0.72 | 0.82 | 1.01 | 0.25 | 1.41 | 1.23 | 0.31 | 0.86 | **0.07** | 0.37 | 0.20 | 0.43 | **5.37** | **2.93** |
| L-Homocysteine (234) | 1.39 | 0.54 | 0.60 | 1.02 | 0.43 | 1.11 | 1.90 | 0.59 | 1.15 | 0.74 | 1.47 | 1.24 | 0.64 | 1.28 |
| L-Homoserine (218) | 3.62 | 1.27 | 2.12 | 0.52 | 0.59 | 1.68 | 0.41 | 0.58 | 2.69 | 5.84 | 2.72 | **10.00** | 2.17 | 1.01 |
| Lysine (156) | 0.50 | 0.36 | 0.37 | 1.43 | 0.73 | 1.01 | 3.95 | 2.69 | **0.36** | 0.84 | 1.20 | 0.31 | **2.30** | **3.28** |
| Malate (245,307) | 1.21 | **0.51** | 0.91 | 0.43 | 0.75 | **1.78** | 0.85 | **1.92** | **0.45** | 1.33 | 0.71 | 0.69 | **2.94** | 1.56 |
| Maleicacid (245) | 0.24 | ND | 0.51 | ND | 2.12 | 0.00 | ND | 1.27 | ND | 1.37 | ND | 1.08 | 0.00 | ND |
| Methionine (176) | 0.90 | **0.49** | **0.58** | 0.51 | **0.64** | 1.18 | 1.03 | 1.06 | **0.58** | 0.86 | 0.87 | 0.81 | 1.49 | **1.51** |
| myo-Inositol (305) | 1.01 | 0.67 | 1.53 | 0.87 | 1.52 | 2.29 | 1.31 | 2.73 | 4.60 | 6.82 | 4.52 | 2.49 | 1.48 | 0.98 |
| Myo-Inositol-P (318) | 1.08 | 1.13 | 1.20 | 1.28 | 1.11 | 1.06 | 1.13 | 0.40 | 0.74 | 1.85 | 0.67 | **4.66** | 2.52 | 0.92 |
| N-Acetyl-Aspartate (274) | **2.31** | **3.71** | **11.68** | 0.57 | **5.05** | **3.15** | **0.15** | **4.82** | **19.18** | **61.43** | 1.11 | **12.73** | **3.20** | **0.06** |
| N-Acetyl-L-Serine (261) | 3.06 | 1.02 | 0.89 | 2.48 | **0.29** | 0.87 | 2.42 | 1.20 | 1.47 | 1.18 | 1.25 | 0.98 | 0.80 | 0.85 |
| o-acetyl-L-Homoserine (202) | 1.69 | 0.83 | 0.56 | 0.45 | 0.33 | 0.68 | 0.54 | 1.15 | 0.90 | 1.08 | 1.29 | 0.94 | 1.20 | 1.44 |
| o-acetyl-L-Serine (132) | 1.55 | 0.72 | ND | 6.55 | ND | ND | 9.07 | 3.39 | 1.28 | 1.21 | 0.97 | 0.36 | 0.95 | 0.76 |
| Ornithine (142) | 1.03 | **0.48** | 0.68 | 0.55 | 0.66 | 1.40 | 1.15 | 1.06 | 0.73 | 1.06 | 0.90 | 1.00 | 1.45 | 1.24 |
| Oroticacid (254) | 1.84 | 2.75 | 5.63 | 1.29 | 3.06 | 2.04 | 0.47 | 1.96 | **31.00** | **88.75** | 1.25 | **45.22** | **2.86** | **0.04** |
| Panthotenic acid (201) | 2.28 | 1.11 | 0.97 | 1.43 | **0.42** | 0.87 | 1.29 | 2.35 | 0.93 | 1.10 | 1.11 | **0.47** | 1.18 | 1.19 |
| PEP (369) | 6.36 | 1.34 | 0.48 | ND | 0.08 | 0.36 | ND | 1.15 | 0.53 | 2.14 | 0.25 | 1.86 | 4.05 | 0.47 |

Identified metabolites of the ancestral and evolved strains by gas chromatography-mass spectrometry.

| | | | | | | | | | | | | | | |
|---|---|---|---|---|---|---|---|---|---|---|---|---|---|---|
| Phenylalanine (192) | 2.46 | 1.10 | 2.30 | 1.88 | 0.94 | 2.09 | 1.70 | 1.31 | 1.21 | 2.20 | 1.88 | 1.68 | 1.82 | 1.56 |
| Phosphate19.28 (299) | 0.94 | 0.45 | 0.51 | 5.95 | 0.54 | 1.14 | 13.29 | 0.75 | **14.23** | 1.22 | 4.38 | 1.64 | 0.09 | 0.31 |
| Proline (142) | 1.27 | 0.53 | 1.28 | 0.63 | 1.01 | **2.41** | 1.18 | 1.71 | 0.62 | 1.41 | 1.27 | 0.83 | **2.28** | **2.06** |
| Putrescine (142,174) | 0.33 | **0.41** | **0.43** | 0.38 | 1.30 | 1.04 | 0.92 | 0.70 | 0.95 | 0.91 | 1.12 | 1.29 | 0.95 | 1.17 |
| Pyruvate (174) | 1.05 | **0.47** | 0.82 | 0.33 | 0.78 | 1.74 | 0.69 | 1.19 | 0.87 | 1.84 | 0.87 | 1.55 | **2.11** | 0.99 |
| Ribitol | 0.86 | 0.76 | 0.91 | 0.66 | 1.06 | 1.19 | 0.86 | 0.94 | 0.78 | 0.90 | 0.94 | 0.96 | 1.15 | 1.20 |
| Ribose (217) | 0.69 | **0.39** | **0.47** | 1.08 | 0.68 | 1.21 | **2.80** | 0.86 | 0.42 | 0.90 | 1.05 | 1.05 | 2.14 | 2.49 |
| Ribose-5-P (315,299) | 1.04 | **0.55** | 0.69 | 0.80 | 0.66 | 1.24 | 1.44 | 1.08 | 0.68 | 1.13 | 0.86 | 1.04 | 1.65 | 1.26 |
| Salicylicacid (267) | 1.03 | **0.43** | 0.72 | 0.40 | 0.70 | 1.67 | 0.92 | 1.51 | **0.48** | 1.21 | 0.57 | 0.80 | **2.55** | 1.19 |
| Serine (204) | 1.22 | 0.65 | 0.92 | 0.53 | 0.75 | 1.41 | 0.81 | 1.45 | 0.84 | 1.26 | 1.20 | 0.87 | 1.50 | 1.42 |
| Shikimate (204) | 0.42 | 0.51 | **0.38** | 0.11 | 0.91 | 0.75 | 0.22 | 0.52 | 0.55 | 0.37 | 0.11 | 0.72 | 0.68 | 0.21 |
| Spermidine (144) | 1.05 | 0.67 | **0.60** | 0.93 | 0.58 | 0.90 | 1.39 | 0.83 | 0.94 | 0.93 | 0.92 | 1.12 | 0.99 | 0.98 |
| Spermine (144) | 0.93 | 0.81 | 1.01 | 1.10 | 1.09 | 1.25 | 1.36 | 1.24 | 1.15 | 0.90 | 0.88 | 0.72 | 0.78 | 0.77 |
| Succinate (247,409) | 0.97 | 0.45 | 0.97 | 0.85 | 1.01 | 2.17 | 1.89 | 1.72 | 0.64 | 1.31 | 1.08 | 0.76 | **2.03** | 1.68 |
| Sucrose (361) | 0.85 | 0.71 | 0.78 | 0.78 | 0.92 | 1.10 | 1.10 | 1.06 | 0.77 | 0.89 | 0.77 | 0.84 | 1.15 | 1.00 |
| Threonine (101) | **2.97** | 0.92 | 1.62 | 0.71 | 0.54 | 1.75 | 0.77 | 0.60 | 0.70 | 4.26 | 2.33 | **7.12** | 6.07 | 3.32 |
| Thymine (255) | **0.53** | 0.63 | 0.66 | 0.47 | 1.23 | 1.03 | 0.74 | **0.67** | **0.36** | **0.55** | **0.69** | 0.82 | 1.51 | **1.91** |
| Trehalose (361) | **2.39** | **2.00** | **6.63** | 0.59 | 2.77 | **3.32** | **0.29** | **2.01** | 0.85 | **3.97** | **0.53** | 1.98 | 4.69 | 0.63 |
| Tryptophan (202) | 0.77 | 0.71 | 0.82 | 0.67 | 1.05 | 1.14 | 0.94 | 0.72 | 0.65 | 0.56 | 0.67 | 0.77 | 0.86 | 1.04 |
| Tyrosine (218) | 1.46 | 1.05 | 0.69 | 1.67 | 0.47 | 0.66 | 1.59 | 0.92 | 3.58 | 0.62 | 1.03 | 0.67 | 0.17 | 0.29 |
| Unknown14.80 (228) | 1.09 | 0.73 | 0.82 | 0.26 | 0.75 | 1.12 | 0.36 | 1.09 | **0.32** | 1.44 | 0.66 | 1.31 | **4.46** | **2.06** |
| Unknown32.96 (361) | **183.88** | 1.32 | **231.43** | 1.88 | 1.26 | **175.44** | 1.42 | **196.78** | **4.59** | **255.29** | 1.43 | 1.30 | **55.62** | 0.31 |
| Uracil (255,241) | 1.09 | **0.46** | 1.03 | 0.70 | 0.95 | **2.24** | 1.50 | 1.42 | **0.49** | **1.88** | 1.11 | 1.33 | **3.83** | **2.27** |
| Urea (189) | 0.60 | 0.06 | 0.17 | 0.06 | 0.29 | 2.81 | 0.99 | 1.43 | 0.30 | 0.25 | 0.44 | 0.18 | 0.84 | 1.46 |
| Valine (144) | 0.36 | 0.66 | **0.53** | 0.53 | 1.47 | 0.80 | 0.81 | 0.99 | **0.54** | **0.44** | 0.64 | **0.44** | 0.81 | 1.19 |
