# Supplementary material for: Perceiving molecular evolution processes in Escherichia coli by comprehensive metabolite and gene expression profiling
Source: Genome Biol. 2008 Apr 10;9(4):R72. doi: 10.1186/gb-2008-9-4-r72 (PMC2643943; doi:10.1186/gb-2008-9-4-r72)
Supplement: Additional data file 2 — Presented is a table listing significantly altered metabolites in all of the evolved conditions. [file gb-2008-9-4-r72-S2.pdf]

Significantly changed metabolites (SAM score (*d*) ≥ 1.0 (or) ≤ -1.0) in excess-nutrient adaptive evolutionary condition.					
Over abundant metabolites in both the lines (MGAdp & DHAdp)			Less abundant metabolites in both the lines (MGAdp & DHAdp)		
**Metabolites**	**Score(*d*)**	**Fold Change**	**Metabolites**	**Score(*d*)**	**Fold Change**
5-Methyl-thioadenosine (236)	3.16	2.35	Erythrose-4-P (357)	-1.27	0.22
Adenine (264)	3.70	2.12	N-Acetyl_Aspartate (274)	-4.65	0.27
a-Glycerophosphate (357)	2.21	1.53	Oroticacid (254)	-1.80	0.36
a-Ketoglutarate(198)	1.57	5.03	Unknown30.70 (217)	-1.22	0.52
Alanine (116)	2.17	1.83			
Arginine (256)	2.22	1.91			
Asparagine (216)	2.55	3.03			
Cystathionine (128)	5.56	5.16			
Glucose-6-P (387)	1.44	1.34			
Glutamate (230,246)	2.31	2.01			
Glycerate-2-P (299,315,459)	2.81	1.72			
Glycine (174)	1.25	1.39			
Lysine (156)	1.42	2.77			
Malate (245,307)	3.33	1.96			
Methionine (176)	3.17	2.03			
o-acetyl_L_Homoserin3 (128)	1.50	1.41			
Ornithine (142)	3.91	2.08			
Proline (142)	1.75	1.88			
Ribose (217)	4.78	2.60			
Ribose-5-P (315,299)	2.77	1.81			
Salicylicacid (267)	2.34	2.31			
Succinate (247,409)	1.96	2.22			
Thymine (255)	2.08	1.57			
Tryptophane (202)	1.97	1.40			
Uracil (255,241)	3.32	2.16			
Urea (189)	1.14	16.14			
Valine (144)	1.35	1.52			

Significantly changed metabolites (SAM score (*d*) ≥ 1.0 (or) ≤ -1.0) in environmental shift evolutionary condition.					
Over abundant metabolites in both the lines (MGAdpGal & DHAdpGal)			Less abundant metabolites in both the lines (MGAdpGal & DHAdpGal)		
**Metabolites**	**Score(*d*)**	**Fold Change**	**Metabolites**	**Score(*d*)**	**Fold Change**
2-Isopropylmalate (275)	2.01	1.68	N-Acetyl_Aspartate (274)	-2.52	0.20
Agmantine (347)	2.13	1.60	Trehalose (361)	-1.90	0.36
Cystathionine (128)	2.25	3.23	Oroticacid (254)	-1.89	0.33
Malate (245,307)	1.41	1.34			
Panthotenic_acid (201)	3.10	2.35			
Putrescine (174)	2.27	1.79			
Urea (189)	0.98	3.43			

**Significantly changed metabolites (SAM score (*d*) ≥ 1.0 (or) ≤ -1.0) in prolonged stationary phase evolutionary condition.**					
Over abundant metabolites in both the lines (MGStat & DHStat)			Less abundant metabolites in both the lines (MGStat & DHStat)		
**Metabolites**	**Score(*d*)**	**Fold Change**	**Metabolites**	**Score(*d*)**	**Fold Change**
Adenine (264)	1.76	2.42	Arabinose (217)	-1.74	0.64
a-Ketoglutarate (198)	1.62	6.46	Erythrose-4-P (357)	-1.30	0.21
Leucine (158)	1.64	3.93	Phenylalanine (192)	-1.02	0.53
Malate (245,307)	2.04	2.31	phosphate19.28 (299)	-2.22	0.17
Proline (142)	1.52	2.88	Trehalose (361)	-1.73	0.48
Salicylicacid (267)	1.76	2.50	Unknown32.96 (361)	-1.41	0.53
Shikimate (204)	1.00	8.82	Unknown44.66 (325)	-1.68	0.30
Thymine (255)	1.73	2.12			
Trehalose (361)	1.74	1.71			
Tryptophan (202)	1.17	1.50			
Urea (189)	1.15	16.34			
Valine (144)	1.60	1.88			
