# Supplementary material for: Perceiving molecular evolution processes in Escherichia coli by comprehensive metabolite and gene expression profiling
Source: Genome Biol. 2008 Apr 10;9(4):R72. doi: 10.1186/gb-2008-9-4-r72 (PMC2643943; doi:10.1186/gb-2008-9-4-r72)
Supplement: Additional data file 3 — Presented is a table listing significantly altered genes in all of the evolved conditions. [file gb-2008-9-4-r72-S3.pdf]

A) Significantly expressed genes (SAM score (*d*) ≥ 1.0 (or) ≤ -1.0) in excess-nutrient adaptive evolutionary condition.

Positive significant genes (SAM score (*d*) ≥ 1.0), i.e. over-expressed genes in both the excess-nutrient evolved lines (MGAdp and DHAdp).				
**B Number**	**Gene Name**	**Function**	**Score (*d*)**	**Fold Change**
B0012	htgA	htgA,transcriptional activator for sigma H (sigma 32) promoters, permitting growth at high temperature	1.46	1.09
B0101	yacG	yacG,conserved hypothetical protein	2.15	1.37
B0104	guaC	guaC,GMP reductase	1.05	1.37
B0135	yadC	yadC,putative fimbrial-like adhesin protein	1.00	2.15
B0178	hlpA	hlpA,periplasmic molecular chaperone for outer membrane proteins	1.19	1.93
B0249	ykfF	ykfF,CP4-6 prophage	0.98	1.18
B0293	yagZ	yagZ,conserved hypothetical protein	1.49	1.38
B0475	hemH	hemH,ferrochelatase	1.26	1.40
B0528	ybcJ	ybcJ,putative RNA-binding protein	1.03	1.40
B0553	nmpC	nmpC,DLP12 prophage; outer membrane porin, at locus of qsr prophage	0.96	1.21
B0564	appY	appY,DLP12 prophage; transcriptional regulator required for anaerobic and stationary phase induction of genes	0.99	1.20
B0571	cusR	cusR,response regulator in two-component regulatory system with CusS, regulation of copper resistance	2.03	1.15
B0603	ybdO	ybdO,putative transcriptional regulator with periplasmic binding protein domain (LysR familiy)	0.98	1.50
B0649	ybeV	ybeV,putative chaperone with dnaJ-like domain	1.82	1.36
B0687	seqA	seqA,negative modulator of replication initiation	0.99	1.52
B0723	sdhA	sdhA,succinate dehydrogenase, catalytic and NAD/flavoprotein subunit	1.13	1.19
B0732	ybgG	ybgG,putative sugar hydrolase with alpha-mannosidase domain	1.27	1.77
B0823	pflF	pflF,putative pyruvate formate lyase	4.06	1.46
B0851	nfsA	nfsA,oxygen-insensitive NADPH nitroreductase, also anaerobic azo reductase	1.46	1.52
B0856	potH	potH,putrescine transport protein (ABC superfamily, membrane)	2.84	2.08
B0904	focA	focA,formate transport protein (formate channel 1) (FNT family)	0.98	1.54
B0906	ycaP	ycaP,conserved hypothetical protein	1.43	1.63
B0958	sulA	sulA,suppressor of lon; inhibitor of cell division and FtsZ ring formation upon DNA damage/inhibition	1.11	1.31
B0996	torC	torC,cytochrome c-type protein in TMAO respiration; with TorA, also negative regulator of tor operon	1.19	1.15
B1006	ycdG	ycdG,putative uracil transport protein (NCS2 family)	1.22	1.36
B1046	ymdC	ymdC,putative synthase with phospholipase D/nuclease domain	1.97	1.31
B1110	ycfJ	ycfJ,putative membrane protein	1.12	1.23
B1114	mfd	mfd,transcription-repair ATP-dependent coupling factor	2.49	1.29

B1173	b1173	b1173,unknown CDS	1.72	1.26
B1183	umuD	umuD,component of DNA polymerase V, signal peptidase with UmuC	1.43	1.66
B1185	dsbB	dsbB,disulfide bond formation proteins (oxidoreductase) with quinone as electron acceptor, reoxidizes DsbA	0.99	1.46
B1194	ycgR	ycgR,putative regulator (TetR/AcrR family)	1.86	1.24
B1216	chaA	chaA,sodium/calcium:proton antiporter (CaCA family)	1.29	1.21
B1354	b1354	b1354,Rac prophage	2.23	1.33
B1372	stfR	stfR,Rac prophage; putative tail fiber protein	1.04	1.22
B1382	ynbE	ynbE,unknown CDS	1.40	1.33
B1483	ddpF	ddpF,putative dipeptide transport protein (ABC superfamily, atp_bind)	1.95	1.36
B1501	ydeP	ydeP,putative formate dehydrogenase, related to acid resistance with formate dehydrogenase/DMSO reductase	1.42	1.26
B1535	ydeH	ydeH,conserved protein	1.89	2.45
B1586	ynfD	ynfD,conserved hypothetical protein	1.05	1.14
B1600	ydgF	ydgF,multidrug transport protein (SMR superfamily)	1.57	1.22
B1601	b1601	b1601,putative integral membrane transport protein	0.99	1.22
B1650	nemA	nemA,N-ethylmaleimide reductase, FMN-linked	2.03	1.25
B1687	ydiJ	ydiJ,putative oxidase, with FAD-binding domains	1.70	1.30
B1689	ydiL	ydiL,conserved hypothetical protein	1.60	1.39
B1693	aroD	aroD,3-dehydroquinate dehydratase	1.25	1.33
B1713	pheT	pheT,phenylalanine tRNA synthetase, beta-subunit	4.92	1.31
B1747	astA	astA,arginine succinyltransferase	1.29	1.21
B1750	ydjX	ydjX,putative membrane protein	2.92	1.30
B1879	flhA	flhA,putative export protein for flagellar biosynthesis	1.02	1.28
B1882	cheY	cheY,chemotactic response regulator in two-component regulatory system with CheA	2.02	1.33
B2040	rfbD	rfbD,TDP-rhamnose synthetase, NAD(P)-binding	4.51	2.24
B2072	b2072	b2072,conserved hypothetical protein	1.08	1.76
B2092	gatC	gatC,PTS family enzyme IIC, galactitol-specific	2.27	2.34
B2125	yehT	yehT,putative response regulator in two-component regulatory system	1.06	1.07
B2208	napF	napF,Fe-S ferredoxin-type protein, electron transfer	1.70	1.45
B2249	b2249	b2249,putative competence-damage protein, related to molybdopterin biosynthesis protein	1.45	1.17
B2251	yfaO	yfaO,putative enzyme (Nudix hydrolase)	5.60	1.86
B2280	nuoJ	nuoJ,NADH dehydrogenase I chain J	1.14	1.10
B2282	nuoH	nuoH,NADH dehydrogenase I chain H	1.11	1.51
B2290	yfbQ	yfbQ,putative PLP-dependent aminotransferase	1.32	1.72
B2356	yfdM	yfdM,CPS-53 (KpLE1) prophage; putative transferase	3.55	1.79
B2363	b2363	b2363,CPS-53 (KpLE1) prophage	2.55	1.96
B2440	eutC	eutC,ethanolamine ammonia-lyase, light chain	1.20	1.77

B2453	eutG	eutG,putative alcohol dehydrogenase with dehydroquinate synthase-like domain, in ethanolamine utilization	1.05	1.49
B2480	bcp	bcp,thiol peroxidase, thioredoxin-dependent	2.92	1.70
B2497	uraA	uraA,uracil transport protein (NCS2 family)	1.71	1.37
B2539	hcaF	hcaF,3-phenylpropionate dioxygenase, beta subunit	1.11	1.51
B2580	ung	ung,uracil-DNA-glycosylase	2.96	1.25
B2581	yfiF	yfiF,putative tRNA/rRNA methyltransferase	1.12	1.60
B2598	pheL	pheL,chorismate mutase-P-prephenate dehydratase leader peptide	2.25	1.33
B2599	pheA	pheA,bifunctional: chorismate mutase P (N-terminal); prephenate dehydratase (C-terminal)	4.50	1.80
B2634	yfjR	yfjR,CP4-57 prophage; putative transcriptional repressor (DeoR family)	2.73	1.40
B2647	ypjA	ypjA,putative outer membrane protein with pectin lyase-like domain	1.19	1.27
B2649	b2649	b2649,conserved protein	1.01	1.33
B2772	b2772	b2772,putative enzyme (synthase) with alpha-macroglobulin receptor domain and FAD-linked C-terminal domain	1.19	1.42
B2805	fucR	fucR,transcriptional activator for L-fucose utilization (DeoR family)	1.33	1.47
B2850	ygeF	ygeF,conserved hypothetical protein	1.79	1.23
B2862	ygeP	ygeP,unknown CDS	1.26	2.10
B2863	b2863	b2863,unknown CDS with six-hairpin glycosyltransferase domain	2.70	1.59
B2866	xdhA	xdhA,putative xanthine dehydrogenase subunit, molybdemum cofactor-binding domain	1.84	1.99
B2955	yggW	yggW,putative oxidase	1.26	1.26
B2991	hybF	hybF,putative hydrogenase expression/formation protein	1.86	1.30
B3019	parC	parC,DNA topoisomerase IV, subunit A	1.12	1.27
B3132	agaZ	agaZ,tagatose 6-phosphate aldolase 1, subunit together with AgaY	1.30	1.29
B3154	yhbP	yhbP,conserved protein	1.30	1.44
B3202	rpoN	rpoN,sigma N (sigma 54) factor of RNA polymerase	1.56	1.28
B3203	yhbH	yhbH,putative sigma N (sigma 54) modulator	2.83	2.00
B3217	yhcE	yhcE,unknown CDS	0.95	1.19
B3299	rpmJ	rpmJ,50S ribosomal subunit protein X	1.02	1.89
B3346	yheO	yheO,putative regulator	1.06	1.34
B3361	fic	fic,possible cell filamentation protein, induced in stationary phase	1.13	1.19
B3520	yhjB	yhjB,putative response regulator in two-component regulatory system	0.98	1.25
B3521	yhjC	yhjC,putative transcriptional regulator with periplasmic binding protein domain (LysR family)	1.26	1.25
B3555	yiaG	yiaG,putative transcriptional regulator with DNA-binding domain	1.74	1.39
B3575	yiaK	yiaK,2,3-diketo-L-gulonate dehydrogenase, NADH-dependent	1.30	1.27
B3654	yicE	yicE,putative purine/xanthine transport protein (NCS2 family)	1.00	1.31
B3685	yidE	yidE,putative transport protein	2.40	1.19
B3687	ibpA	ibpA,small heat shock protein	1.62	1.24
B3736	atpF	atpF,membrane-bound ATP synthase, F0 sector, subunit b	1.19	1.77

B3743	asnC	asnC,transcriptional regulator of asparagine biosynthesis (AsnC family)	2.51	1.64
B3745	yieM	yieM,conserved protein with Integrin A (or I) domain	1.01	1.28
B3746	yieN	yieN,putative transcriptional regulator	1.41	1.37
B3753	rbsR	rbsR,transcriptional repressor for ribose metabolism (GalR/LacI family)	1.02	1.55
B3886	rbn	rbn,tRNA processing exoribonuclease BN	2.47	1.59
B3923	yiiT	yiiT,putative stress flavoprotein with ETFP electron transfer domain	1.00	1.26
B3966	btuB	btuB,outer membrane porin: cobalamin transport, receptor for E colicins, bacteriophage BF23	1.68	1.60
B4065	yjcE	yjcE,putative sodium:hydrogen antiporter (CPA1 family)	1.42	1.38
B4137	cutA	cutA,periplasmic divalent cation tolerance protein; cytochrome c biogenesis	1.54	1.70
B4146	yjeK	yjeK,putative aminomutase	0.98	1.14
B4216	ytfJ	ytfJ,conserved hypothetical protein	1.17	1.16
B4267	idnD	idnD,L-idonate 5-dehydrogenase, NAD-binding	1.01	1.19
B4305	sgcX	sgcX,putative endoglucanase with Zn-dependent exopeptidase domain	1.08	1.26
B4330	yjiH	yjiH,putative membrane protein	1.25	1.28
B4355	tsr	tsr,methyl-accepting chemotaxis protein I, serine sensor receptor	0.97	1.23
B4395	gpmB	gpmB,putative phosphoglyceromutase 2	0.98	1.20

Negative significant genes (SAM score (*d*) ≤ 1.0), i.e. under-expressed genes in both the excess-nutrient evolved lines (MGAdp and DHAdp).				
**B Number**	**Gene Name**	**Function**	**Score (*d*)**	**Fold Change**
B0015	dnaJ	dnaJ,heat shock protein (Hsp40), co-chaperone with DnaK	-0.97	0.80
B0155	yadQ	yadQ,putative chlorine transport protein (ClC family)	-1.01	0.77
B0396	araJ	araJ,arabinose polymer transport protein (MFS family)	-1.15	0.76
B0491	ybbM	ybbM,putative transport protein, metal resistance protein (YbbM family)	-1.61	0.79
B0497	rhsD	rhsD,RhsD protein in RhsD element	-1.49	0.73
B0550	rus	rus,DLP12 prophage; endodeoxyribonuclease RUS (Holliday junction resolvase)	-2.23	0.51
B0563	ylcE	ylcE,DLP12 prophage	-0.99	0.90
B0577	ybdG	ybdG,putative transport protein	-1.29	0.79
B0589	fepG	fepG,ferric enterobactin transport protein (ABC superfamily, membrane)	-1.49	0.84
B0624	crcB	crcB,Crc interacts with Csp affecting chromosome condensation	-1.69	0.87
B0647	ybeT	ybeT,conserved hypothetical protein	-1.56	0.64

B0651	rihA	rihA,ribonucleoside hydrolase 1, pyrimidine-specific	-1.05	0.73
B0713	ybgL	ybgL,putative lactam utilization protein	-1.48	0.61
B0737	tolQ	tolQ,inner membrane protein required for outer membrane integrity, uptake of group A colicins and phage DNA	-1.27	0.81
B0762	ybhT	ybhT,unknown CDS	-3.65	0.53
B0778	bioD	bioD,dethiobiotin synthetase	-1.80	0.88
B0781	moaA	moaA,molybdopterin biosynthesis protein A	-6.34	0.55
B0848	ybjM	ybjM,putative membrane protein	-1.62	0.82
B0876	ybjD	ybjD,conserved protein with nucleotide triphosphate hydrolase domain	-2.63	0.47
B0908	aroA	aroA,3-enolpyruvylshikimate-5-phosphate synthetase	-1.02	0.81
B0998	torD	torD,cytoplasmic chaperone involved in maturation of TorA	-1.04	0.77
B1033	ycdW	ycdW,2-ketoacid reductase	-2.46	0.69
B1035	ycdY	ycdY,conserved protein	-1.03	0.74
B1063	yceB	yceB,conserved hypothetical protein	-1.30	0.65
B1073	flgB	flgB,flagellar biosynthesis; cell-proximal portion of basal-body rod	-1.40	0.54
B1114	mfd	mfd,transcription-repair ATP-dependent coupling factor	-1.00	0.70
B1195	ymgE	ymgE,conserved hypothetical protein	-1.37	0.70
B1201	dhaR	dhaR,putative transcriptional regulator in two-component regulatory system	-1.11	0.80
B1223	narK	narK,nitrite extrusion protein (MFS family)	-1.63	0.43
B1232	purU	purU,formyltetrahydrofolate hydrolase	-1.61	0.69
B1249	cls	cls,cardiolipin synthase	-1.47	0.74
B1270	btuR	btuR,cob(I)alamin and cobinamide ATP-dependent adenolsyltransferase	-5.69	0.19
B1271	yciK	yciK,putative oxoacyl-(acyl carrier protein) reductase with NAD(P)-binding domain	-4.01	0.37
B1272	sohB	sohB,putative peptidase	-8.67	0.25
B1276	acnA	acnA,aconitate hydratase 1	-3.51	0.46
B1292	sapC	sapC,peptide transport protein (ABC superfamily, membrane)	-1.09	0.69
B1335	ogt	ogt,O-6-alkylguanine-DNA/cysteine-protein methyltransferase	-1.53	0.76
B1346	ydaQ	ydaQ,Rac prophage	-1.16	0.58
B1369	b1369	b1369,Rac prophage; putative outer membrane protein	-1.24	0.70
B1432	b1432	b1432,putative transposase	-2.01	0.87
B1514	ydeY	ydeY,putative sugar transport protein (ABC superfamily, membrane)	-1.71	0.76
B1530	marR	marR,transcriptional repressor for antibiotic resistance and oxidative stress	-1.34	0.64
B1573	ydfC	ydfC,Qin prophage	-1.34	0.72
B1607	ydgC	ydgC,putative membrane protein	-2.25	0.63
B1622	malY	malY,bifunctional: PLP-dependent beta-cystathionase; repressor of maltose regulon through interaction with MalT	-1.19	0.73
B1640	ydhH	ydhH,conserved hypothetical protein with actin-like ATPase domain	-1.08	0.63
B1664	b1664	b1664,putative enzyme with pectin lyase-like domain	-5.42	0.45

B1673	ydhV	ydhV,putative aldehyde ferridoxin oxidoreductase	-1.51	0.83
B1767	ansA	ansA,cytoplasmic L-asparaginase I	-1.38	0.85
B1822	rrmA	rrmA,23S rRNA m1G745 methyltransferase	-1.44	0.87
B1885	tap	tap,methyl-accepting chemotaxis protein IV, peptide sensor receptor	-1.86	0.77
B1889	motB	motB,enables flagellar motor rotation, linking torque machinery to cell wall	-1.42	0.84
B1913	uvrC	uvrC,DNA exisiton repair enzyme together with UvrAB	-1.15	0.61
B1929	yedE	yedE,putative membrane component of transport system	-1.80	0.43
B1930	yedF	yedF,conserved hypothetical protein with SirA-like domain	-0.97	0.55
B1968	yedV	yedV,putative sensory kinase in regulatory system	-2.81	0.55
B2012	yeeD	yeeD,conserved hypothetical protein with SirA-like domain	-1.99	0.73
B2016	yeeZ	yeeZ,putative enzyme with NAD(P)-binding domain	-1.79	0.75
B2105	yohL	yohL,conserved hypothetical protein	-0.96	0.82
B2127	mlrA	mlrA,transcriptional regulator of curli and extracellular matrix synthesis (MerR family)	-1.15	0.60
B2153	folE	folE,GTP cyclohydrolase I	-1.25	0.73
B2156	lysP	lysP,lysine-specific permease (APC family)	-1.12	0.68
B2199	ccmC	ccmC,heme transfer protein, cytochrome c-type biogenesis	-1.25	0.77
B2316	accD	accD,acetylCoA carboxylase, carboxyltranferase subunit beta	-3.23	0.75
B2320	pdxB	pdxB,erythronate-4-phosphate dehydrogenase	-1.29	0.85
B2443	b2443	b2443,CPZ-55 prophage	-1.42	0.53
B2491	hyfR	hyfR,transcriptional activator for expression of hydrogenase 4 genes, interacts with sigma 54 (EBP family)	-1.22	0.89
B2524	yfhJ	yfhJ,putative protein believed to be involved in assembly of Fe-S clusters	-1.35	0.59
B2526	hscA	hscA,chaperone (Hsp70 family), believed to be involved in assembly of Fe-S clusters	-1.25	0.84
B2593	yfiH	yfiH,conserved hypothetical protein	-1.58	0.60
B2627	yfjK	yfjK,CP4-57 prophage	-1.02	0.80
B2703	srlE	srlE,PTS family enzyme IIBC, glucitol/sorbitol-specific	-1.29	0.67
B2717	hycI	hycI,protease involved in processing C-terminal end of HycE	-1.10	0.73
B2734	pphB	pphB,serine/threonine-specific protein phosphatase 2	-1.03	0.65
B2811	ygdK	ygdK,conserved hypothetical protein	-1.45	0.75
B2827	thyA	thyA,thymidylate synthetase	-2.12	0.80
B2829	ptsP	ptsP,PTS family enzyme I, transcriptional regulator (with NPR and NTR proteins)	-2.16	0.51
B2845	b2845	b2845,putative transport protein (HAAAP family)	-1.68	0.42
B2848	yqeJ	yqeJ,conserved hypothetical protein	-1.26	0.76
B2883	ygfP	ygfP,guanine deaminase	-1.21	0.83
B2896	ygfX	ygfX,conserved hypothetical protein	-1.66	0.73
B2915	yqfE	yqfE,conserved hypothetical protein with periplasmic binding protein II-like domain	-1.09	0.83
B2930	yggF	yggF,putative enzyme	-1.10	0.85

B2950	yggR	yggR,putative transport protein with P-loop containing nucloside triphosphate hydrolase domain	-2.79	0.66
B2951	yggS	yggS,putative enzyme with PLP-binding domain	-1.30	0.74
B3038	ygiC	ygiC,putative glutathione-like synthetase	-1.98	0.67
B3059	ygiH	ygiH,putative membrane protein	-1.31	0.54
B3067	rpoD	rpoD,sigma D (sigma 70) factor of RNA polymerase	-0.97	0.84
B3068	mug	mug,DNA glycosylase, G/U mismatch-specific	-1.28	0.83
B3104	yhaI	yhaI,putative membrane protein	-2.11	0.66
B3108	yhaM	yhaM,conserved hypothetical protein	-1.04	0.63
B3108	yhaM	yhaM,conserved hypothetical protein	-1.18	0.59
B3111	tdcGa	tdcGa,L-serine dehydratase 3, part 2, fuses with b3112, one orf	-1.71	0.66
B3115	tdcD	tdcD,propionate kinase/acetate kinase C, anaerobic	-2.93	0.39
B3124	garK	garK,glycerate kinase I	-0.97	0.67
B3212	gltB	gltB,glutamate synthase, large subunit	-1.11	0.64
B3213	gltD	gltD,glutamate synthase, small subunit, nucleotide-binding, 4Fe-4S protein	-1.69	0.57
B3230	rpsI	rpsI,30S ribosomal subunit protein S9	-2.73	0.59
B3242	yhcR	yhcR,unknown CDS	-1.13	0.70
B3248	yhdE	yhdE,conserved hypothetical protein with Maf/Ham1 domain	-1.46	0.63
B3280	yrdB	yrdB,conserved hypothetical protein	-1.22	0.70
B3284	smg	smg,conserved hypothetical protein	-1.10	0.48
B3291	mscL	mscL,mechanosensitive channel	-6.05	0.20
B3292	zntR	zntR,Zn(II)-responsive transcriptional regulator, regulates Zn export (MerR family)	-4.62	0.27
B3303	rpsE	rpsE,30S ribosomal subunit protein S5	-3.83	0.48
B3307	rpsN	rpsN,30S ribosomal subunit protein S14	-1.30	0.75
B3312	rpmC	rpmC,50S ribosomal subunit protein L29	-1.29	0.61
B3347	fkpA	fkpA,FKBP-type peptidyl-prolyl cis-trans isomerase (rotamase)	-1.64	0.55
B3355	prkB	prkB,putative phosphoribulokinase with nucleoside triP hydrolase domain	-1.49	0.59
B3406	greB	greB,transcription elongation factor and transcript cleavage	-1.81	0.43
B3415	gntT	gntT,high-affinity gluconate permease in GNT I system (GntP family)	-1.10	0.76
B3438	gntR	gntR,transcriptional repressor for gluconate utilization (GalR/LacI familiy)	-1.09	0.91
B3498	prlC	prlC,oligopeptidase A	-2.18	0.60
B3506	slp	slp,outer membrane protein, induced after carbon starvation	-2.12	0.75
B3597	yibH	yibH,putative membrane protein	-1.38	0.84
B3610	grxC	grxC,glutaredoxin 3	-0.97	0.83
B3621	rfaC	rfaC,ADP-heptose; LPS heptosyl transferase I	-1.12	0.81
B3647	ligB	ligB,DNA ligase	-1.53	0.59
B3673	emrD	emrD,multidrug transport protein (MFS family)	-1.02	0.77

B3676	yidH	yidH,conserved hypothetical protein	-1.76	0.72
B3699	gyrB	gyrB,DNA gyrase, subunit B (type II topoisomerase)	-0.97	0.85
B3710	yidY	yidY,drug/chloramphenicol transport protein (MFS family)	-1.18	0.58
B3742	mioC	mioC,FMN-binding protein, required for biotin synthase activity	-1.00	0.60
B3824	rhtB	rhtB,homoserine/homoserine lactone efflux protein (RhtB family)	-1.70	0.57
B3828	metR	metR,transcriptional regulator of methionine biosynthesis (LysR family)	-1.22	0.65
B3904	rhaB	rhaB,rhamnulokinase	-1.26	0.74
B3912	cpxR	cpxR,response regulator in two-component regulatory system with CpxA	-1.31	0.74
B3917	sbp	sbp,sulfate transport protein (ABC superfamily, peri_bind)	-1.67	0.60
B3927	glpF	glpF,MIP channel, glycerol diffusion	-1.39	0.78
B3954	yijO	yijO,putative transcriptional regulator with homeodomain-like DNA binding domain	-1.33	0.63
B3962	sthA	sthA,soluble pyridine nucleotide transhydrogenase	-2.95	0.55
B3982	nusG	nusG,component in transcription antitermination	-1.12	0.78
B3988	rpoC	rpoC,RNA polymerase, beta prime subunit	-1.87	0.47
B4011	yjaA	yjaA,conserved hypothetical protein	-1.44	0.73
B4038	yjbI	yjbI,conserved protein	-1.65	0.76
B4054	tyrB	tyrB,tyrosine aminotransferase, tyrosine-repressible, PLP-dependent	-1.47	0.72
B4172	hfq	hfq,host factor I for bacteriophage Q beta replication, RNA binding, stimulates poly(A) polymerase I	-1.35	0.76
B4188	yjfN	yjfN,conserved hypothetical protein	-1.81	0.73
B4193	sgaT	sgaT,PTS family enzyme IIC, ascorbate-specific	-1.60	0.51
B4220	ytfM	ytfM,putative outer membrane protein	-1.45	0.79
B4247	yjgG	yjgG,unknown CDS	-1.53	0.79
B4285	b4285	b4285,KpLE2 phage-like element; putative transposase	-2.31	0.43
B4290	fecB	fecB,KpLE2 phage-like element; citrate-dependent iron (III) transport protein (ABC superfamily, peri_bind)	-1.15	0.61
B4309	yjhS	yjhS,conserved protein	-1.04	0.88
B4311	yjhA	yjhA,conserved hypothetical protein, outer membrane domain	-0.96	0.83
B4313	fimE	fimE,tyrosine recombinase, regulator of fimA	-2.87	0.55
B4331	kptA	kptA,2'-phosphotransferase	-1.11	0.74

B) Significantly expressed genes (SAM score (*d*) ≥ 1.0 (or) ≤ -1.0) in environmental shift evolutionary condition.

Positive significant genes (SAM score (*d*) ≥ 1.0), i.e. over-expressed genes in both the environmental shift evolved lines (MGAdpGal and DHAdpGal).				
**B Number**	**Gene Name**	**Function**	**Score (*d*)**	**Fold Change**
B0444	ybaX	ybaX,putative (aluminum) resistance protein with adenine nucleotide alpha hydrolase domain	1.14	1.35
B0528	ybcJ	ybcJ,putative RNA-binding protein	0.96	1.37
B1354	b1354	b1354,Rac prophage	1.46	1.41
B1689	ydiL	ydiL,conserved hypothetical protein	2.44	1.55
B3203	yhbH	yhbH,putative sigma N (sigma 54) modulator	1.35	1.39

Negative significant genes (SAM score (*d*) ≤ 1.0), i.e. under-expressed genes in both the environmental shift evolved lines (MGAdpGal and DHAdpGal).				
**B Number**	**Gene Name**	**Function**	**Score (*d*)**	**Fold Change**
B0291	yagX	yagX,putative aromatic compound dioxygenase	-1.21	0.72
B0422	xseB	xseB,exonuclease VII, small subunit	-0.99	0.85
B1271	yciK	yciK,putative oxoacyl-(acyl carrier protein) reductase with NAD(P)-binding domain	-1.18	0.68
B1272	sohB	sohB,putative peptidase	-2.51	0.29
B1351	racC	racC,Rac prophage; contains recE and oriJ	-0.99	0.81
B1445	ydcX	ydcX,unknown CDS	-1.49	0.76
B1464	yddE	yddE,conserved protein	-1.60	0.74
B1467	narY	narY,nitrate reductase 2, beta subunit	-1.04	0.79
B1683	sufB	sufB,putative transport protein associated with Fe-S cluster assembly	-1.28	0.73
B1735	celD	celD,transcriptional repressor of carbon uptake (AraC/XylS family)	-1.26	0.82
B1820	b1820	b1820,conserved protein	-1.04	0.82
B2829	ptsP	ptsP,PTS family enzyme I, transcriptional regulator (with NPR and NTR proteins)	-0.95	0.77
B2941	yqgD	yqgD,unknown CDS	-1.23	0.75
B3038	ygiC	ygiC,putative glutathione-like synthetase	-2.37	0.55
B3056	cca	cca,tRNA nucleotidyl transferase	-1.27	0.78

B3067	rpoD	rpoD,sigma D (sigma 70) factor of RNA polymerase	-1.87	0.66
B3068	mug	mug,DNA glycosylase, G/U mismatch-specific	-1.40	0.71
B3104	yhaI	yhaI,putative membrane protein	-1.78	0.59
B3115	tdcD	tdcD,propionate kinase/acetate kinase C, anaerobic	-2.09	0.50
B3230	rpsI	rpsI,30S ribosomal subunit protein S9	-1.83	0.62
B3291	mscL	mscL,mechanosensitive channel	-2.49	0.34
B3292	zntR	zntR,Zn(II)-responsive transcriptional regulator, regulates Zn export (MerR family)	-2.33	0.37
B3405	ompR	ompR,response regulator in two-component regulatory system with EnvZ, regulates ompF and ompC expression	-1.01	0.80
B3498	prlC	prlC,oligopeptidase A	-1.36	0.73
B3696	yidX	yidX,conserved hypothetical protein	-1.82	0.70
B3697	yidA	yidA,conserved protein, phosphatase-like domain	-1.08	0.79
B3802	hemY	hemY,protein in late step of protoheme IX synthesis with tetratricopeptide repeats (TPR) domain	-1.15	0.81
B4024	lysC	lysC,aspartokinase III, lysine sensitive	-1.51	0.77
B4172	hfq	hfq,host factor I for bacteriophage Q beta replication, RNA binding, mRNA degradation, stimulates poly(A) polymerase I	-1.38	0.72
B4313	fimE	fimE,tyrosine recombinase, regulator of fimA	-2.02	0.65

C) Significantly expressed genes (SAM score (*d*) ≥ 1.0 (or) ≤ -1.0) in prolonged stationary phase evolutionary condition.

Positive significant genes (SAM score (*d*) ≥ 1.0), i.e. over-expressed genes in both the prolonged stationary phase evolved lines (MGStat and DHStat).				
**B Number**	**Gene Name**	**Function**	**Score (*d*)**	**Fold Change**
B0057	yabQ	yabQ,unknown CDS	1.07	1.30
B0142	folK	folK,2-amino-4-hydroxy-6-hydroxymethyldihyropteridine pyrophosphokinase	1.18	1.32
B0260	mmuP	mmuP,CP4-6 prophage; putative S-methylmethionine transport protein (APC family)	2.57	1.94
B0355	yaiM	yaiM,putative esterase	1.15	1.33
B0454	ybaZ	ybaZ,putative methylated DNA-protein cysteine methyltransferase, C-terminal domain	0.97	1.17
B0456	ybaA	ybaA,conserved hypothetical protein	2.06	1.28
B0471	ybaB	ybaB,conserved hypothetical protein	1.01	1.25
B0571	cusR	cusR,response regulator in two-component regulatory system with CusS, regulation of copper resistance	1.48	1.23
B0577	ybdG	ybdG,putative transport protein	2.10	1.63
B0654	gltJ	gltJ,glutamate/aspartate transport protein (ABC superfamily, membrane)	1.14	1.29
B0663	B0663	B0663,putative rna	1.10	1.15
B0723	sdhA	sdhA,succinate dehydrogenase, catalytic and NAD/flavoprotein subunit	1.09	1.29
B0855	potG	potG,putrescine transport protein (ABC superfamily, atp_bind)	1.56	1.17
B0859	rumB	rumB,23S rRNA m(5)U747 methyltransferase	0.98	1.29
B0981	etk	etk,tyrosine-protein kinase ETK, autophosphorylates	1.06	1.31
B1071	flgM	flgM,anti-FliA (anti-sigma) factor; also known as RflB protein	1.17	1.19
B1156	tfaE	tfaE,e14 prophage; putative tail fiber assembly	1.16	1.21
B1168	ycgG	ycgG,conserved protein	1.36	1.52
B1266	trpH	trpH,conserved hypothetical protein	1.16	1.29
B1270	btuR	btuR,cob(I)alamin and cobinamide ATP-dependent adenolsyltransferase	2.11	1.82
B1271	yciK	yciK,putative oxoacyl-(acyl carrier protein) reductase with NAD(P)-binding domain	2.07	1.77
B1276	acnA	acnA,aconitate hydratase 1	2.17	1.76
B1285	gem	gem,RNase II modulator with PYP-like sensor domain	1.16	1.27
B1288	fabI	fabI,enoyl-[acyl-carrier-protein] reductase (NADH)	1.67	1.31
B1566	flxA	flxA,Qin prophage	1.63	1.26
B1581	rspA	rspA,putative dehydratase, starvation-sensing protein, member of enolase family	0.98	1.15
B1653	lhr	lhr,putative ATP-dependent helicase, superfamily II with P-loop hydrolase domain and winged helix domain	1.45	1.27
B1664	b1664	b1664,putative enzyme with pectin lyase-like domain	1.88	1.54
B1683	sufB	sufB,putative transport protein associated with Fe-S cluster assembly	1.35	1.28

B1688	ydiK	ydiK,putative transport protein	1.71	1.25
B1940	fliH	fliH,flagellar biosynthesis; putative export of flagellar proteins	1.07	1.32
B2094	gatA	gatA,PTS family enzyme IIA, galactitol-specific	1.03	1.27
B2276	nuoN	nuoN,NADH dehydrogenase I chain N, membrane subunit	1.26	1.36
B2363	b2363	b2363,CPS-53 (KpLE1) prophage	1.24	1.43
B2542	hcaD	hcaD,phenylpropionate dioxygenase, ferredoxin reductase subunit	1.09	1.16
B2555	yfhG	yfhG,conserved protein	1.02	1.18
B2562	yfhL	yfhL,putative 4Fe-4S ferredoxin-type protein	1.07	1.39
B2585	pssA	pssA,phosphatidylserine synthase (CDP-diacylglycerol-serine O-phosphatidyltransferase)	1.22	1.15
B2761	ygcB	ygcB,putative enzyme with 4 nucleoside triP hydrolase domains	2.14	1.49
B2763	cysI	cysI,sulfite reductase, beta subunit, NADPH-dependent hemoprotein	1.82	1.22
B2769	ygcQ	ygcQ,putative electron transfer flavoprotein, NAD/FAD-binding domain	1.00	1.28
B2879	ssnA	ssnA,putative protein with metallo-dependent hydrolase domain, commonly a deaminase	1.05	1.09
B3063	ygjE	ygjE,putative tartrate:succinate antiporter (DASS family)	0.99	1.18
B3067	rpoD	rpoD,sigma D (sigma 70) factor of RNA polymerase	1.58	1.28
B3068	mug	mug,DNA glycosylase, G/U mismatch-specific	1.35	1.27
B3261	fis	fis,DNA-binding protein for site-specific recombination and inversion	1.14	1.27
B3291	mscL	mscL,mechanosensitive channel	1.10	3.23
B3292	zntR	zntR,Zn(II)-responsive transcriptional regulator, regulates Zn export (MerR family)	3.69	2.82
B3348	slyX	slyX,conserved hypothetical protein	1.34	1.33
B3548	yhjY	yhjY,putative lipase	1.06	1.26
B3570	bax	bax,conserved hypothetical protein	1.68	1.36
B3652	recG	recG,DNA helicase, ATP-dependent resolution of Holliday junctions, branch migration	1.05	1.08
B3744	asnA	asnA,asparagine synthetase A	1.35	1.29
B3783	rho	rho,transcription termination factor Rho; polarity suppressor	1.39	1.25
B3786	wecB	wecB,UDP-N-acetyl glucosamine-2-epimerase	1.30	1.29
B3788	rffG	rffG,dTDP-glucose 4,6-dehydratase	1.40	1.36
B3860	dsbA	dsbA,periplasmic protein disulfide isomerase I, disulfide bond formation	1.15	1.26
B3894	fdoG	fdoG,formate dehydrogenase-O, major subunit	1.39	1.35
B4088	alsB	alsB,allose transport protein (ABC superfamily, peri_bind)	1.45	1.22
B4147	efp	efp,elongation factor P (EF-P)	2.33	1.36
B4156	yjeM	yjeM,putative amino-acid transport protein (APC family)	2.52	1.36
B4159	yjeP	yjeP,putative periplasmic binding protein	1.70	1.34
B4168	yjeE	yjeE,putative enzyme with nucleoside triP hydrolase domain	1.07	1.18
B4170	mutL	mutL,enzyme in methyl-directed mismatch repair, stimulates binding of Vsr and MutS to heteroduplex DNA	2.21	1.55
B4172	hfq	hfq,host factor I for bacteriophage Q beta replication, RNA binding, stimulates poly(A) polymerase I	1.88	1.42
B4181	yjfI	yjfI,conserved hypothetical protein	1.09	1.17

B4201	priB	priB,primosomal replication protein N	1.85	1.51
B4206	ytfB	ytfB,conserved protein	1.87	1.33
B4292	fecR	fecR,KpLE2 phage-like element; membrane-bound regulator in multi-component regulatory system	1.32	1.28

Negative significant genes (SAM score (*d*) ≤ 1.0), i.e. under-expressed genes in both the prolonged stationary phase evolved lines (MGStat and DHStat).				
**B Number**	**Gene Name**	**Function**	**Score (*d*)**	**Fold Change**
B0208	yafC	yafC,putative transcriptional regulator with periplasmic binding protein domain (LysR family)	-1.27	0.80
B0479	fsr	fsr,fosmidomycin/multidrug transport protein (MFS family)	-0.96	0.71
B0483	ybaQ	ybaQ,putative repressor with DNA-binding domain	-1.41	0.80
B0602	ybdN	ybdN,conserved protein with adenine nucleotide alpha hydrolase domain	-1.02	0.87
B0721	sdhC	sdhC,succinate dehydrogenase, hydrophobic subunit, cytochrome b556 with SdhD	-1.39	0.73
B0781	moaA	moaA,molybdopterin biosynthesis protein A	-1.79	0.76
B0814	ompX	ompX,outer membrane protease, receptor for phage OX2	-1.86	0.72
B1099	holB	holB,DNA polymerase III, delta prime subunit	-1.56	0.76
B1216	chaA	chaA,sodium/calcium:proton antiporter (CaCA family)	-0.98	0.84
B1235	hnr	hnr,response regulator involved in protein turnover, controls stability of RpoS	-1.03	0.86
B1312	ycjP	ycjP,putative transport protein (ABC superfamily, membrane)	-2.42	0.68
B1389	paaB	paaB,putative subunit of multicomponent oxygenase, phenylacetic acid degradation	-0.95	0.84
B1490	yddV	yddV,conserved protein	-1.42	0.81
B1601	b1601	b1601,putative integral membrane transport protein	-0.97	0.83
B1689	ydiL	ydiL,conserved hypothetical protein	-1.11	0.79
B1695	ydiO	ydiO,putative acyl-CoA dehydrogenase (flavoprotein)	-1.57	0.81
B1839	b1839	b1839,conserved hypothetical protein	-1.23	0.79
B2024	hisA	hisA,N-(5'-phospho-L-ribosyl-formimino)-5-amino-1-(5'-phosphoribosyl)-4-imidazolecarboxamide isomerase	-1.34	0.80
B2107	b2107	b2107,unknown CDS	-2.28	0.72
B2133	dld	dld,D-lactate dehydrogenase, NADH-independent, FAD-binding domain	-1.14	0.77
B2503	b2503	b2503,putative oxidative protein	-1.20	0.80
B2537	hcaR	hcaR,transcriptional activator of hca cluster (LysR family)	-0.97	0.79
B2565	recO	recO,gap repair protein, part of RecFOR complex that targets RecA to ssDNA-dsDNA junction	-1.25	0.77
B2690	yqaB	yqaB,putative phosphoglucomutase, contains a phosphatase-like domain	-2.01	0.73
B2697	alaS	alaS,alanyl-tRNA synthetase	-1.15	0.86
B2701	mltB	mltB,membrane-bound lytic murein transglycosylase B	-1.17	0.77

B2802	fucI	fucI,L-fucose isomerase	-0.96	0.80
B2808	gcvA	gcvA,transcriptional regulator (postitive) of cleavage of glycine (LysR family)	-1.38	0.75
B2809	ygdI	ygdI,unknown CDS	-1.92	0.74
B2819	recD	recD,exonuclease V, alpha chain with recC and recD: 5' and 3' nuclease, ATPase, recombinase, helicase	-1.55	0.85
B2829	ptsP	ptsP,PTS family enzyme I, transcriptional regulator (with NPR and NTR proteins)	-1.82	0.64
B2895	fldB	fldB,flavodoxin 2	-1.04	0.87
B2898	ygfZ	ygfZ,putative enzyme	-1.55	0.70
B2930	yggF	yggF,putative enzyme	-1.13	0.84
B2937	speB	speB,agmatinase	-1.71	0.78
B2999	b2999	b2999,putative enzyme with alpha/beta-hydrolase-like domain	-0.98	0.89
B3015	ygiQ	ygiQ,conserved protein	-1.04	0.80
B3136	agaS	agaS,putative tagatose-6-phosphate ketose/aldose isomerase with phosphosugar-binding domain	-1.14	0.80
B3211	yhcC	yhcC,putative enzyme	-1.43	0.84
B3494	uspB	uspB,universal stress protein B	-1.04	0.79
B3510	hdeA	hdeA,conserved protein with protein HNS-dependent expression A; HdeA-like domain	-1.91	0.77
B3516	gadX	gadX,transcriptional regulator for glutamic acid decarboylase and transporter (gadA, gad BC) (AraC/XylS family)	-2.22	0.87
B3698	yidB	yidB,conserved hypothetical protein	-2.63	0.76
B3913	cpxP	cpxP,periplasmic repressor of cpx regulon by interaction with CpxA	-1.35	0.76
B4013	metA	metA,homoserine transsuccinylase	-1.20	0.83
B4053	alr	alr,alanine racemase 1, PLP-binding, biosynthetic	-1.08	0.76
B4080	yjcP	yjcP,putative outer membrane protein	-3.24	0.77
B4108	phnA	phnA,putative alkylphosphonate uptake protein in phosphonate metabolism	-0.98	0.85
B4115	adiC	adiC,arginine:amgatine antiporter (APC family)	-1.10	0.82
B4321	gntP	gntP,gluconate transport protein, GNT III system (GntP family)	-1.40	0.82
B4338	yjiP	yjiP,putative transposase	-2.90	0.69
