# Supplementary material for: Perceiving molecular evolution processes in Escherichia coli by comprehensive metabolite and gene expression profiling
Source: Genome Biol. 2008 Apr 10;9(4):R72. doi: 10.1186/gb-2008-9-4-r72 (PMC2643943; doi:10.1186/gb-2008-9-4-r72)
Supplement: Additional data file 5 — Presented is a figure showing the integration of transcriptome and metabolome data during the comparison of ancestral and evolved strains in excess nutrient adaptive evolution. [file gb-2008-9-4-r72-S5.pdf]

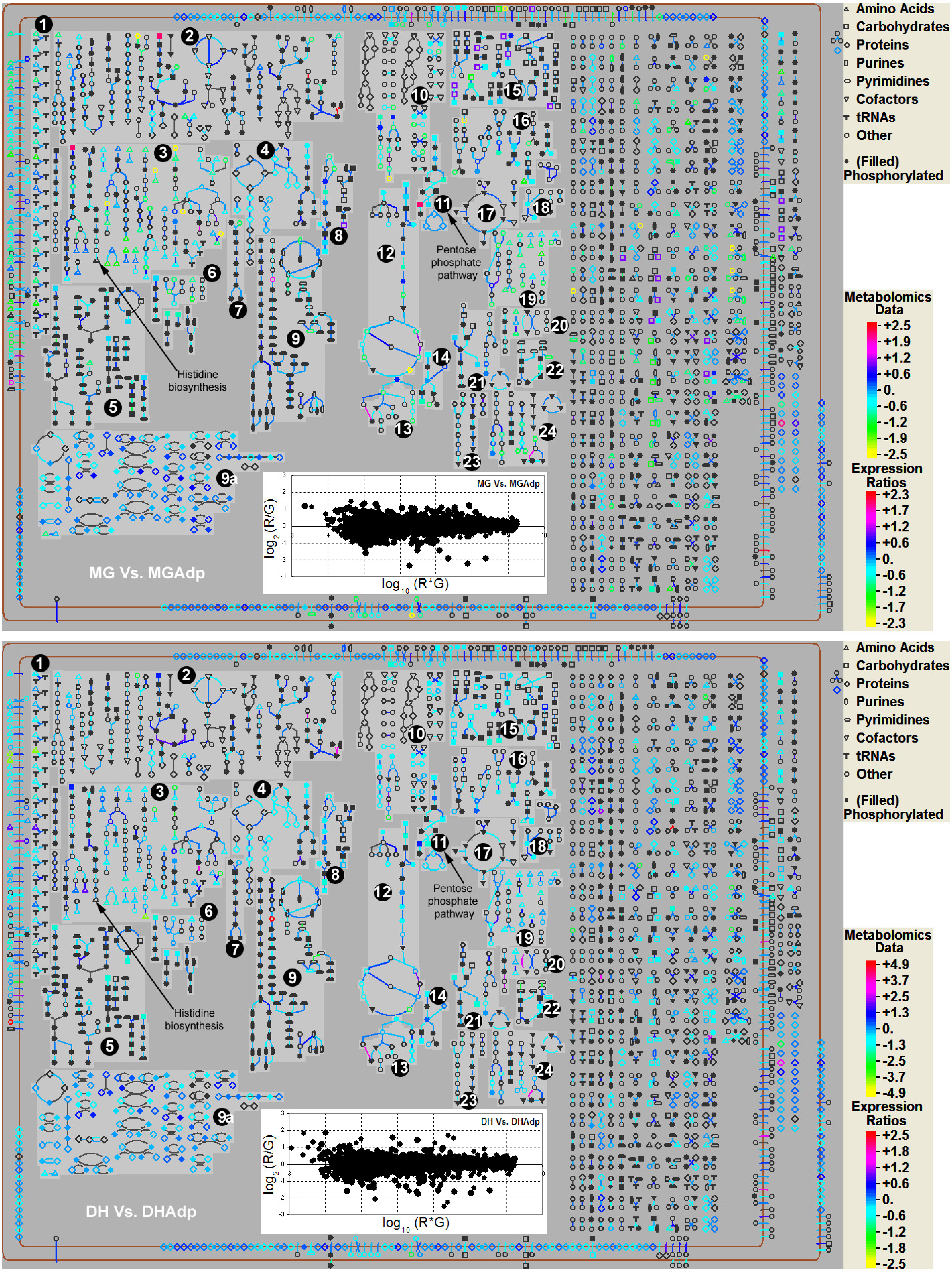
Amino Acids
Carbohydrates
Proteins
Purines
Pyrimidines
Cofactors
tRNAs
Other
(Filled) Phosphorylated
Histidine biosynthesis
Pentose phosphate pathway
MG Vs. MGAdp
MG Vs. MGAdp
log2 (R/G)
log10 (R*G)
Metabolomics Data
+2.5
+1.9
+1.2
+0.6
0.
-0.6
-1.2
-1.9
-2.5
Expression Ratios
+2.3
+1.7
+1.2
+0.6
0.
-0.6
-1.2
-1.7
-2.3
Amino Acids
Carbohydrates
Proteins
Purines
Pyrimidines
Cofactors
tRNAs
Other
(Filled) Phosphorylated
Histidine biosynthesis
Pentose phosphate pathway
DH Vs. DHAdp
DH Vs. DHAdp
log2 (R/G)
log10 (R*G)
Metabolomics Data
+4.9
+3.7
+2.5
+1.3
0.
-1.3
-2.5
-3.7
-4.9
Expression Ratios
+2.5
+1.8
+1.2
+0.6
0.
-0.6
-1.2
-1.8
-2.5

Additional data file 5

Integration of transcriptome and metabolome data during the comparison of ancestral and evolved strains in excess-nutrient adaptive evolution (MG/MGAdp and DH/DHAdp). Metabolic overview for *E. coli* illustrating the results of high-throughput transcriptome and metabolome experiments in a global metabolic pathway context generated by Pathway Tools. Gene expression levels are mapped to reaction steps involved in a metabolism, and the range of data values levels in a given experimental dataset is mapped to a spectrum of colours according to the $\log_2$ ratio scale on the lower right inset. Similarly, for metabolomics data, compound nodes are coloured according to the data value for the corresponding compound. The inner inset shows the ratio-intensity plot of the gene expression data displaying the $\log_2$(Ri/Gi) ratio for each element on the array as a function of the $\log_{10}$(Ri*Gi) product intensities. (1- Biosynthesis - Aminoacyl-tRNAs, 2- Biosynthesis - Cofactors, prosthetic group and electron carriers, 3- Biosynthesis - Amino acids, 4- Biosynthesis - Fattyacids and lipids, 5- Biosynthesis - Cell structure, 6- Biosynthesis - Amines and polyamines, 7- Biosynthesis - Secondary metabolites, 8- Biosynthesis - Sugars and polysaccharides, 9- Biosynthesis - Nucleosides and nucleotides, 9a- Biosynthesis -Signal transduction pathways, 10- Respiration, 11- Pentose phosphate pathway, 12- Glycolysis, 13- Fermentation, 14- Superpathway of glycolysis and Entner-Doudoroff, 15- Degradation - Sugars and polysaccharides, 16- Degradation - Sugar derivatives, 17- Degradation - Fattyacids and lipids, 18- Degradation - Alcohols, 19- Degradation - Amino acids, 20- Degradation - Inorganic nutrients, 21- Degradation - Amines and polyamines, 22- Degradation - Nucleosides and nucleotides, 23- Degradation - Aromatic compounds, 24- Degradation - Carboxylates.)
