# Supplementary material for: Perceiving molecular evolution processes in Escherichia coli by comprehensive metabolite and gene expression profiling
Source: Genome Biol. 2008 Apr 10;9(4):R72. doi: 10.1186/gb-2008-9-4-r72 (PMC2643943; doi:10.1186/gb-2008-9-4-r72)
Supplement: Additional data file 6 — Presented is a figure showing the gene expression and metabolite abundance level in the pentose phosphate pathway in excess nutrient adapted strains. [file gb-2008-9-4-r72-S6.pdf]

Superpathway of oxidative and non-oxidative brances of pentose phosphate pathway

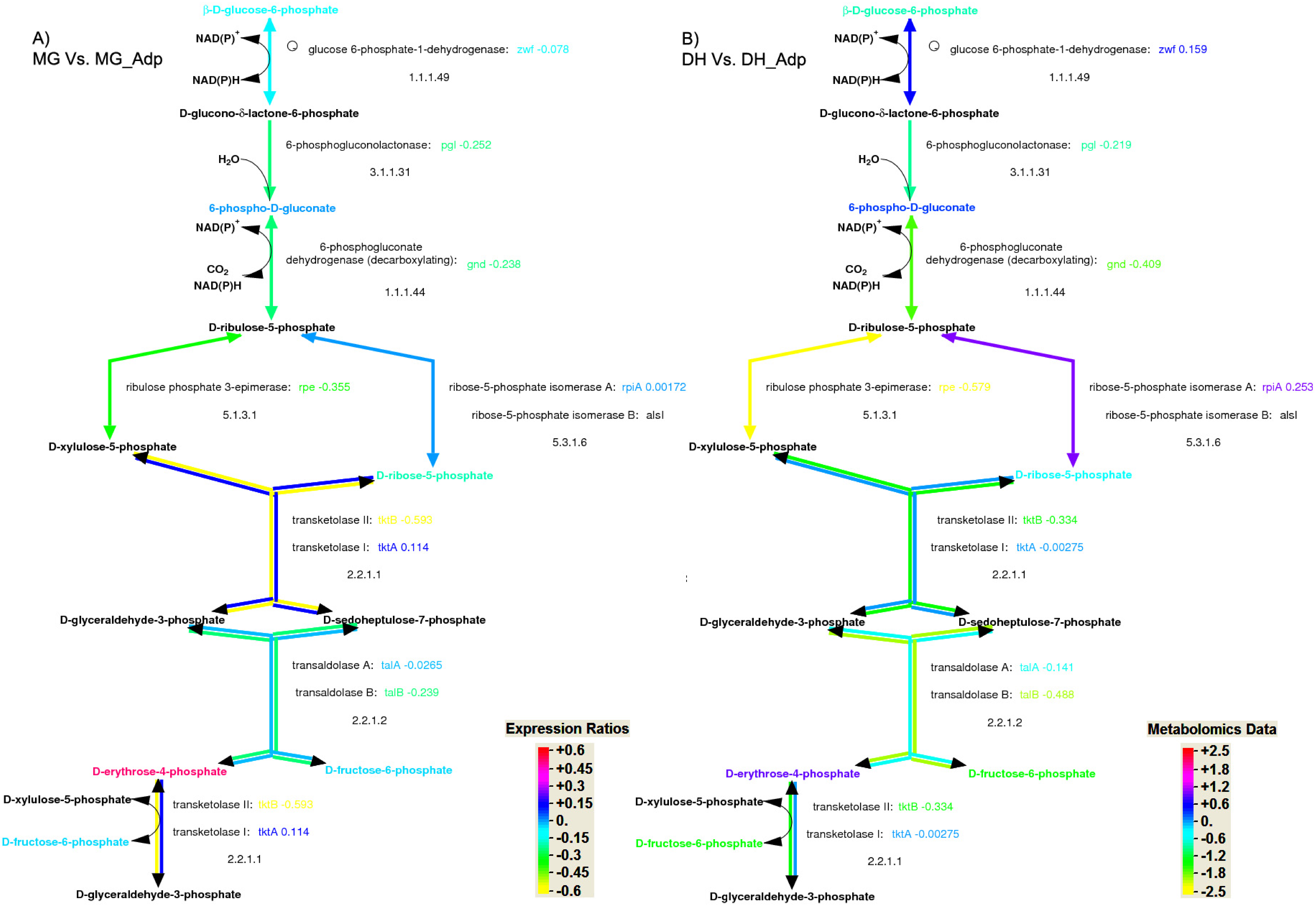

Additional data file 6

Pentose phosphate pathway in excess-nutrient adapted strains. The gene expression and metabolite abundance level in evolved strains compared to their ancestor lines are shown according to the $\log_2$ ratio scale shown in the inset (A- MG/MGAdp, B- DH/DHAdp). Reaction lines are colour-coded according to the relative values of the expression level of the gene that codes for the enzyme that catalyzes that reaction step. Metabolite names are colour-coded according to the relative abundance of the metabolite.
