# Supplementary material for: Perceiving molecular evolution processes in Escherichia coli by comprehensive metabolite and gene expression profiling
Source: Genome Biol. 2008 Apr 10;9(4):R72. doi: 10.1186/gb-2008-9-4-r72 (PMC2643943; doi:10.1186/gb-2008-9-4-r72)
Supplement: Additional data file 7 — Presented is a figure showing PCA analyses for both the ancestor and evolved lines of both strains grown in two different media. [file gb-2008-9-4-r72-S7.pdf]

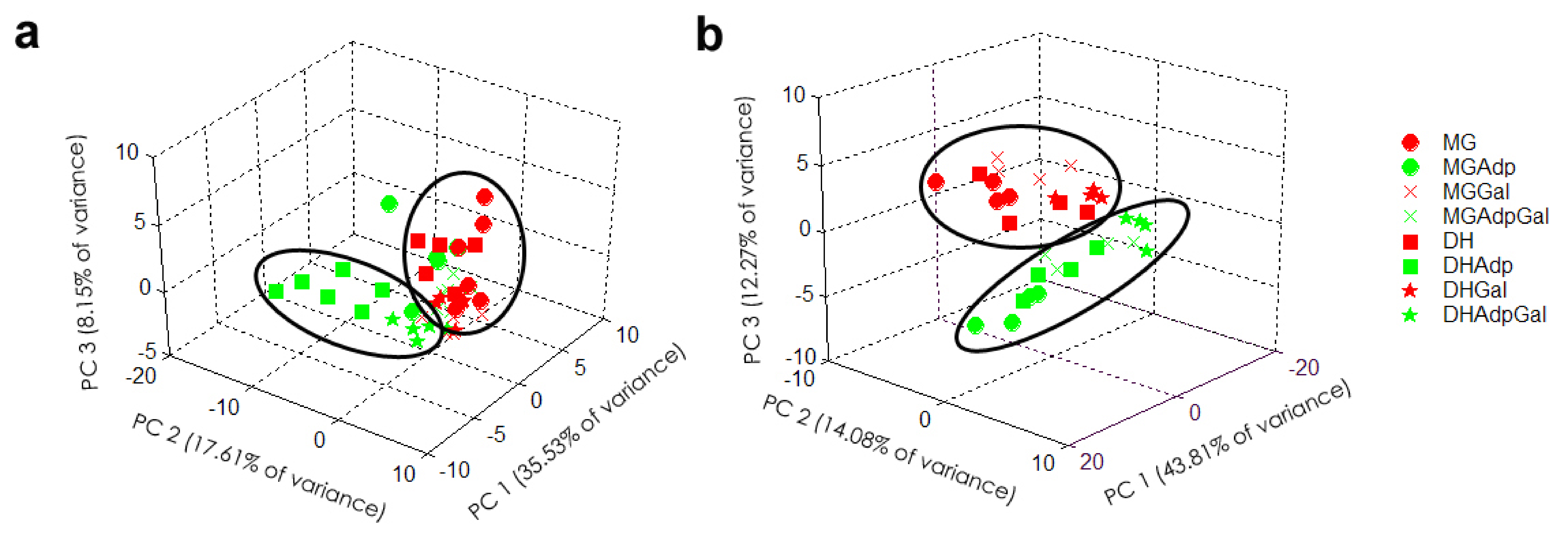
a
PC 3 (8.15% of variance)
PC 2 (17.61% of variance)
PC 1 (35.53% of variance)
b
PC 3 (12.27% of variance)
PC 2 (14.08% of variance)
PC 1 (43.81% of variance)
MG
MGAdp
MGGal
MGAdpGal
DH
DHAdp
DHGal
DHAdpGal

Additional data file 7

PCA analyses of both the ancestors and evolved lines of both the strains grown in two different media. Each data-point represents an experimental sample plotted using the first three principal components. PCA was carried out on the log transformed mean-centred data matrix using all identified metabolites and the genes with $P \leq 0.05$ (Student t-test) in at least one strain. Values given for each component in parentheses represents the percentage of variance.
