# Supplementary material for: Perceiving molecular evolution processes in Escherichia coli by comprehensive metabolite and gene expression profiling
Source: Genome Biol. 2008 Apr 10;9(4):R72. doi: 10.1186/gb-2008-9-4-r72 (PMC2643943; doi:10.1186/gb-2008-9-4-r72)
Supplement: Additional data file 8 — Presented is a table listing common topologic properties of all evolution co-expression networks. [file gb-2008-9-4-r72-S8.pdf]

NETWORK	NODES	EDGES	$\langle k \rangle$		$\langle C \rangle$		$\langle l \rangle$		DIAMETER	
			Coexpression network	Random network	Coexpression network	Random network	Coexpression network	Random network	Coexpression network	Random network
MGDHAdp	4170	23086	11.070	11.070	0.049	0.001	7.193	5.048	20	10
MGDHAdpGal	4136	20501	9.910	9.910	0.051	0.001	7.157	5.367	18	12
MGDHStat	4166	54028	25.930	25.930	0.058	0.002	6.548	3.544	21	6
MGDHAdp Intersection	4168	27117	13.010	13.010	0.113	0.001	6.753	4.665	14	9
MGDHAdpGal Intersection	4129	36976	17.910	17.910	0.070	0.001	6.847	4.037	16	7
MGDHStat Intersection	4141	85095	41.090	41.090	0.089	0.003	6.866	3.028	23	5
