# Supplementary material for: Perceiving molecular evolution processes in Escherichia coli by comprehensive metabolite and gene expression profiling
Source: Genome Biol. 2008 Apr 10;9(4):R72. doi: 10.1186/gb-2008-9-4-r72 (PMC2643943; doi:10.1186/gb-2008-9-4-r72)
Supplement: Additional data file 9 — Presented is a figure showing the gene expression and metabolite abundance level in histidine biosynthesis pathway in excess nutrient adapted strains. [file gb-2008-9-4-r72-S9.pdf]

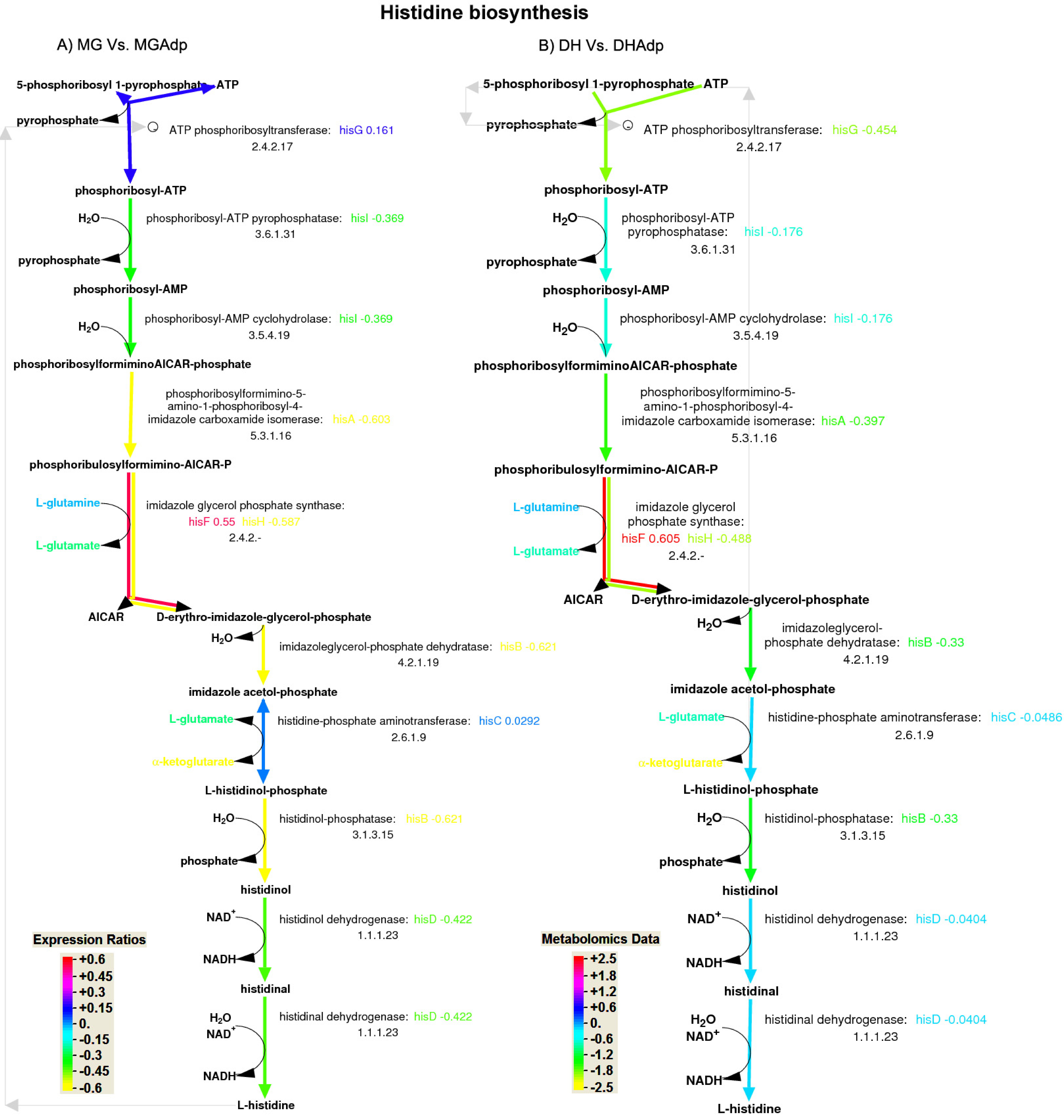

Histidine biosynthesis
A) MG Vs. MGAdp
B) DH Vs. DHAdp
5-phosphoribosyl 1-pyrophosphate
ATP
pyrophosphate
ATP phosphoribosyltransferase: hisG 0.161
2.4.2.17
phosphoribosyl-ATP
H_2O
phosphoribosyl-ATP pyrophosphatase: hisI -0.369
3.6.1.31
pyrophosphate
phosphoribosyl-AMP
H_2O
phosphoribosyl-AMP cyclohydrolase: hisI -0.369
3.5.4.19
phosphoribosylformiminoAICAR-phosphate
phosphoribosylformimino-5-amino-1-phosphoribosyl-4-imidazole carboxamide isomerase: hisA -0.603
5.3.1.16
phosphoribulosylformimino-AICAR-P
L-glutamine
imidazole glycerol phosphate synthase:
hisF 0.55 hisH -0.587
2.4.2.-
L-glutamate
AICAR
D-erythro-imidazole-glycerol-phosphate
H_2O
imidazoleglycerol-phosphate dehydratase: hisB -0.621
4.2.1.19
imidazole acetol-phosphate
L-glutamate
histidine-phosphate aminotransferase: hisC 0.0292
2.6.1.9
α-ketoglutarate
L-histidinol-phosphate
H_2O
histidinol-phosphatase: hisB -0.621
3.1.3.15
phosphate
histidinol
NAD^+
histidinol dehydrogenase: hisD -0.422
1.1.1.23
NADH
histidinal
H_2O
NAD^+
histidinal dehydrogenase: hisD -0.422
1.1.1.23
NADH
L-histidine
Expression Ratios
+0.6
+0.45
+0.3
+0.15
0.
-0.15
-0.3
-0.45
-0.6
5-phosphoribosyl 1-pyrophosphate
ATP
pyrophosphate
ATP phosphoribosyltransferase: hisG -0.454
2.4.2.17
phosphoribosyl-ATP
H_2O
phosphoribosyl-ATP pyrophosphatase: hisI -0.176
3.6.1.31
pyrophosphate
phosphoribosyl-AMP
H_2O
phosphoribosyl-AMP cyclohydrolase: hisI -0.176
3.5.4.19
phosphoribosylformiminoAICAR-phosphate
phosphoribosylformimino-5-amino-1-phosphoribosyl-4-imidazole carboxamide isomerase: hisA -0.397
5.3.1.16
phosphoribulosylformimino-AICAR-P
L-glutamine
imidazole glycerol phosphate synthase:
hisF 0.605 hisH -0.488
2.4.2.-
L-glutamate
AICAR
D-erythro-imidazole-glycerol-phosphate
H_2O
imidazoleglycerol-phosphate dehydratase: hisB -0.33
4.2.1.19
imidazole acetol-phosphate
L-glutamate
histidine-phosphate aminotransferase: hisC -0.0486
2.6.1.9
α-ketoglutarate
L-histidinol-phosphate
H_2O
histidinol-phosphatase: hisB -0.33
3.1.3.15
phosphate
histidinol
NAD^+
histidinol dehydrogenase: hisD -0.0404
1.1.1.23
NADH
histidinal
H_2O
NAD^+
histidinal dehydrogenase: hisD -0.0404
1.1.1.23
NADH
L-histidine
Metabolomics Data
+2.5
+1.8
+1.2
+0.6
0.
-0.6
-1.2
-1.8
-2.5

Additional data file 9

Histidine biosynthesis pathway in excess-nutrient adapted strains. The gene expression level and the metabolite abundance level in the evolved strains compared to their ancestor lines are shown according to the log_2 ratio scale shown in the inset (A- MG/MGAdp, B- DH/DHAdp). The reaction lines are colour-coded according to the relative values of the expression level of the gene that codes for the enzyme that catalyzes that reaction step. Metabolite names are colour-coded according to the relative abundance of the metabolite.
