# Supplementary material for: Perceiving molecular evolution processes in Escherichia coli by comprehensive metabolite and gene expression profiling
Source: Genome Biol. 2008 Apr 10;9(4):R72. doi: 10.1186/gb-2008-9-4-r72 (PMC2643943; doi:10.1186/gb-2008-9-4-r72)
Supplement: Additional data file 10 — Presented is a figure showing the integration of transcriptome and metabolome data during the comparison of ancestral and evolved strains in prolonged stationary phase evolution. [file gb-2008-9-4-r72-S10.pdf]

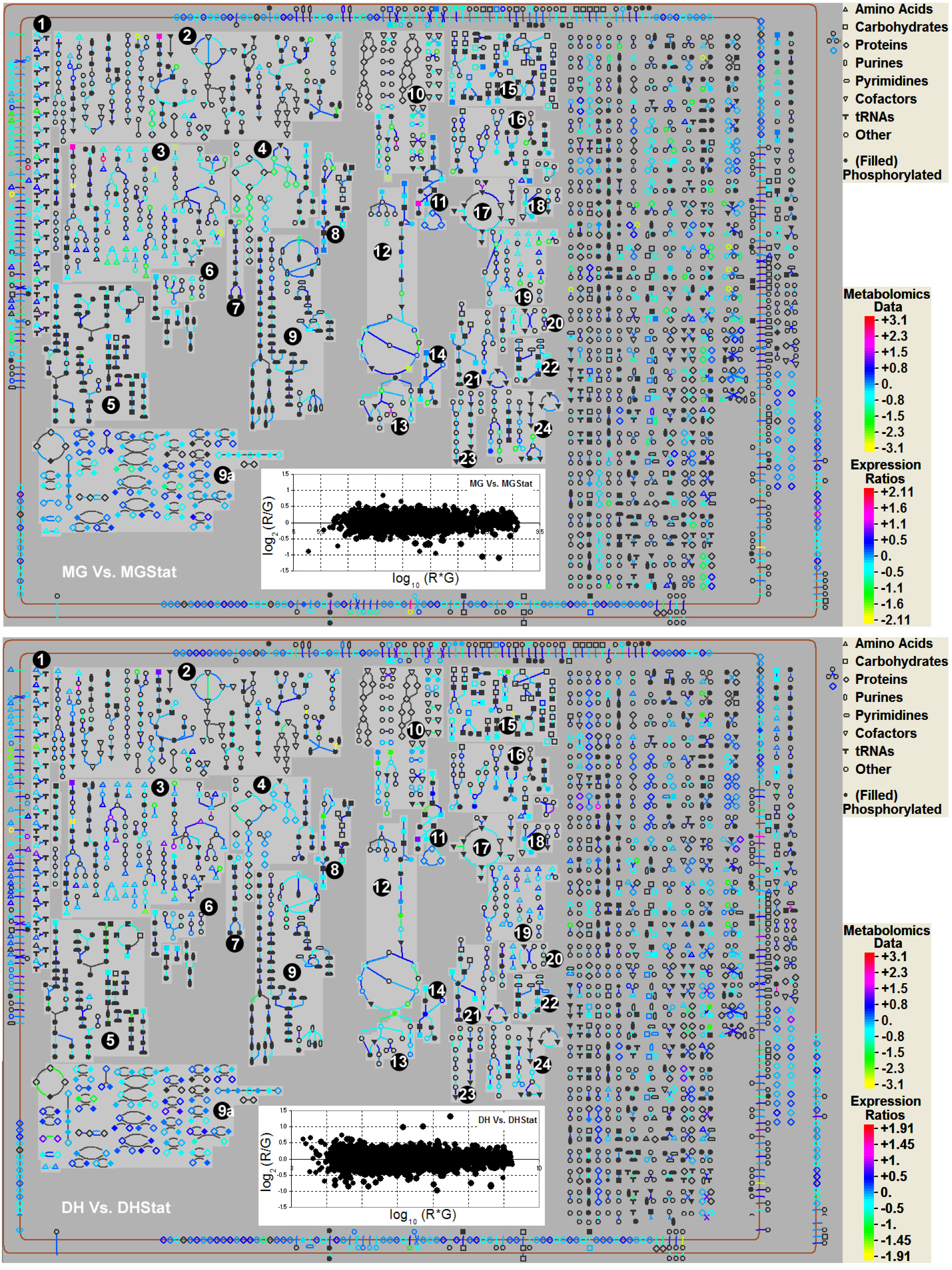
Amino Acids
Carbohydrates
Proteins
Purines
Pyrimidines
Cofactors
tRNAs
Other
(Filled) Phosphorylated
Metabolomics Data
+3.1
+2.3
+1.5
+0.8
0.
-0.8
-1.5
-2.3
-3.1
Expression Ratios
+2.11
+1.6
+1.1
+0.5
0.
-0.5
-1.1
-1.6
-2.11
MG Vs. MGStat
MG Vs. MGStat
log2 (R/G)
log10 (R*G)
1
2
3
4
5
6
7
8
9
9a
10
11
12
13
14
15
16
17
18
19
20
21
22
23
24
Amino Acids
Carbohydrates
Proteins
Purines
Pyrimidines
Cofactors
tRNAs
Other
(Filled) Phosphorylated
Metabolomics Data
+3.1
+2.3
+1.5
+0.8
0.
-0.8
-1.5
-2.3
-3.1
Expression Ratios
+1.91
+1.45
+1.
+0.5
0.
-0.5
-1.
-1.45
-1.91
DH Vs. DHStat
DH Vs. DHStat
log2 (R/G)
log10 (R*G)

**Additional data file 10**

Integration of transcriptome and metabolome data during the comparison of ancestral and evolved strains in prolonged stationary phase evolution (MG/MGStat and DH/DHStat). Metabolic overview for *E. coli* illustrating the results of high-throughput transcriptome and metabolome experiments in a global metabolic pathway context generated by Pathway Tools. Gene expression levels are mapped to reaction steps involved in a metabolism, and the range of data values levels in a given experimental dataset is mapped to a spectrum of colours according to the $\log_2$ ratio scale on the lower right inset. Similarly, for metabolomics data, compound nodes are coloured according to the data value for the corresponding compound. The inner inset shows the ratio-intensity plot of the gene expression data displaying the $\log_2$(Ri/Gi) ratio for each element on the array as a function of the $\log_{10}$(Ri*Gi) product intensities. (1- Biosynthesis - Aminoacyl-tRNAs, 2- Biosynthesis - Cofactors, prosthetic group and electron carriers, 3- Biosynthesis - Amino acids, 4- Biosynthesis - Fattyacids and lipids, 5- Biosynthesis - Cell structure, 6- Biosynthesis - Amines and polyamines, 7- Biosynthesis - Secondary metabolites, 8- Biosynthesis - Sugars and polysaccharides, 9- Biosynthesis - Nucleosides and nucleotides, 9a- Biosynthesis -Signal transduction pathways, 10- Respiration, 11- Pentose phosphate pathway, 12- Glycolysis, 13- Fermentation, 14- Superpathway of glycolysis and Entner-Doudoroff, 15- Degradation - Sugars and polysaccharides, 16- Degradation - Sugar derivatives, 17- Degradation - Fattyacids and lipids, 18- Degradation - Alcohols, 19- Degradation - Amino acids, 20- Degradation - Inorganic nutrients, 21- Degradation - Amines and polyamines, 22- Degradation - Nucleosides and nucleotides, 23- Degradation - Aromatic compounds, 24- Degradation - Carboxylates.)
