# Supplementary material for: Perceiving molecular evolution processes in Escherichia coli by comprehensive metabolite and gene expression profiling
Source: Genome Biol. 2008 Apr 10;9(4):R72. doi: 10.1186/gb-2008-9-4-r72 (PMC2643943; doi:10.1186/gb-2008-9-4-r72)
Supplement: Additional data file 11 — Presented is a figure showing metabolite abundance level and gene expression level among the biologic replicates. [file gb-2008-9-4-r72-S11.pdf]

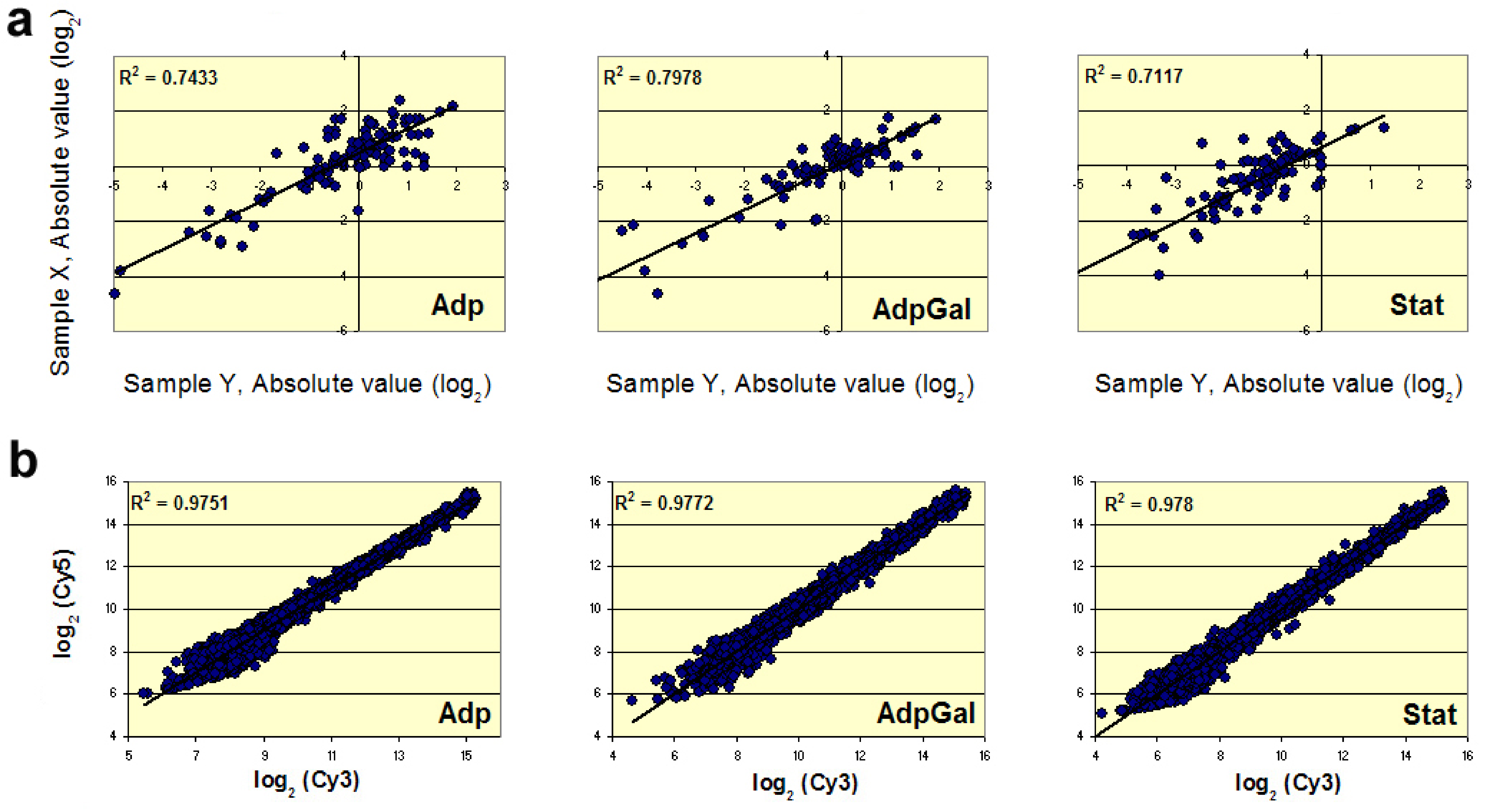
a
b
Sample X, Absolute value (log2)
Sample Y, Absolute value (log2)
R2 = 0.7433
Adp
R2 = 0.7978
AdpGal
R2 = 0.7117
Stat
log2 (Cy5)
log2 (Cy3)
R2 = 0.9751
Adp
R2 = 0.9772
AdpGal
R2 = 0.978
Stat

Additional data file 11

Metabolite abundance level and gene expression level among the biological replicates. The metabolite abundance (a) and gene expression levels (b) among the evolved strain biological replicates indicating relatively low level of variation among the replicates used for the evolutionary studies.
