# Supplementary material for: Perceiving molecular evolution processes in Escherichia coli by comprehensive metabolite and gene expression profiling
Source: Genome Biol. 2008 Apr 10;9(4):R72. doi: 10.1186/gb-2008-9-4-r72 (PMC2643943; doi:10.1186/gb-2008-9-4-r72)
Supplement: Additional data file 4 — Presented is a table listing significant GO functional categories involved in all of the evolved conditions. [file gb-2008-9-4-r72-S4.pdf]

Gene expression changes in experimental evolutionary conditions.

Significant (Wilcoxon rank sum test; *P* ≤ 0.05 shown in bold) functional categories involved in excess-nutrient adaptive evolutionary condition.

**Bin**	**Functional annotation**	**No of Genes**	**MG/ MGAdp**	**No of Genes**	**DH/ DHAdp**
1.2	Macromolecule degradation > GO:macromolecule catabolism ; GO:0009057	105	**0.045**	105	0.312
1.3	Energy metabolism (carbon) > GO:energy derivation by oxidation of organic compounds ; GO:0015980	299	**0.012**	299	0.920
2.1	DNA related > GO:DNA metabolism ; GO:0006259	163	**0.006**	163	**0.004**
4	Transport > GO:transporter activity ; GO:0005215 > GO:transport ; GO:0006810	1215	0.783	1215	**0.028**
5.1	Cell division > GO:cytokinesis ; GO:0000910	95	**0.035**	95	0.230
6.6	Ribosome > GO:cytosolic ribosome (sensu Bacteria) ; GO:0009281	68	0.098	68	**0.018**
8	extrachromosomal > GO:.	300	0.300	300	**0.027**
8.4	Colicin related > GO:.	9	0.165	9	**0.034**
1.1.2.2	3-phenylpropionate and 3-(3-hydroxyphenyl)propionate degradation > GO:3-phenylpropionate catabolism ; GO:0019380 > GO:3-(3-hydroxy)phenylpropionate catabolism ; GO:0019622	11	**0.049**	11	**0.000**
1.1.3	Amino acids > GO:amino acid catabolism ; GO:0009063	74	0.927	74	**0.034**
1.1.3.5	Glycine cleavage > GO:glycine decarboxylation via glycine cleavage system ; GO:0019464	7	0.611	7	**0.010**
1.1.5.2	Ethanol degradation > GO:ethanol catabolism ; GO:0006068	12	**0.025**	12	0.271
1.2.2	DNA > GO:DNA catabolism ; GO:0006308	26	**0.043**	26	0.068
1.3.9	Entner-Doudoroff pathway > GO:Entner-Doudoroff pathway ; GO:0009255	4	**0.041**	4	0.323
1.5.1.11	Serine > GO:serine biosynthesis ; GO:0006564	6	**0.025**	6	0.080
1.5.1.12	Cysteine > GO:cysteine biosynthesis ; GO:0019344	6	**0.049**	6	0.927
1.5.1.15	Tryptophan > GO:tryptophan biosynthesis ; GO:0000162	13	**0.012**	13	**0.020**
1.5.1.17	Alanine > GO:alanine biosynthesis ; GO:0006523	4	**0.038**	4	0.770
1.5.1.21	Homoserine > GO:homoserine biosynthesis ; GO:0009090	6	0.752	6	**0.010**
1.5.1.4	Proline > GO:proline biosynthesis ; GO:0006561	8	0.752	8	**0.036**
1.5.1.7	Lysine, diaminopimelate > GO:lysine biosynthesis via diaminopimelate ; GO:0009089	16	0.153	16	**0.038**
1.5.3.14	Enterochelin (enterobactin) > GO:enterobactin biosynthesis ; GO:0009239	6	0.589	6	**0.011**
1.5.3.4	Molybdenum (molybdopterin) > GO:Mo-molybdopterin cofactor biosynthesis ; GO:0006777	18	**0.021**	18	0.108
1.5.3.6	Pyridoxine (vitamin B6) > GO:pyridoxine biosynthesis ; GO:0008615	9	0.677	9	**0.036**
1.5.4	Fatty acid and phosphatidic acid > GO:fatty acid biosynthesis ; GO:0006633 ; GO:phosphatidic acid biosynthesis ; GO:0006654	26	**0.005**	26	0.215
1.6.12	Flagella > GO:flagella biogenesis ; GO:0009296	37	0.258	37	**0.043**

1.6.3.2	Core region > GO:lipopolysaccharide core region biosynthesis ; GO:0009244	17	0.952	17	**0.029**
1.6.6	Osmoregulated periplasmic glucan > GO:glucan biosynthesis ; GO:0009250	2	**0.049**	2	0.218
1.7.18	Betaine biosynthesis > GO:betaine biosynthesis ; GO:0006578	4	0.302	4	**0.011**
1.7.3	Pentose phosphate shunt, non-oxidative branch > GO:pentose-phosphate shunt\, non-oxidative branch ; GO:0009052	8	**0.036**	8	**0.019**
2.1.5	DNA degradation > GO:DNA catabolism ; GO:0006308	27	**0.049**	27	0.069
2.2.3	RNA modification > GO:RNA modification ; GO:0009451	53	**0.028**	53	**0.029**
2.3.2	Translation > GO:protein biosynthesis ; GO:0006412	74	0.121	74	**0.024**
2.3.6	Turnover, degradation > GO:proteolysis and peptidolysis ; GO:0006508	10	0.527	10	**0.002**
2.3.8	Ribosomal proteins > GO:structural constituent of ribosome ; GO:0003735	57	0.190	57	**0.017**
3.1.1	DNA structure level > GO:.	14	**0.010**	14	**0.002**
3.1.1.2	Methylation > GO:DNA methylation ; GO:0006306	5	**0.022**	5	0.083
3.1.3.1	Translation attenuation and efficiency > GO:translational attenuation ; GO:0009386	10	**0.024**	10	0.101
3.1.3.4	Proteases, cleavage of compounds > GO:peptidase activity ; GO:0008233	9	0.268	9	**0.036**
4.10.119	methylgalactoside/galactose > GO:galactose transporter activity ; GO:0005354 > GO:galactose transporter activity ; GO:0005354 > GO:methylgalactoside transporter activity ; GO:0015592 > GO:methylgalactoside transport ; GO:0015765	4	0.660	4	**0.049**
4.10.155	phosphate > GO:phosphate transport ; GO:0006817 > GO:phosphate transporter activity ; GO:0015114	7	**0.030**	7	0.643
4.10.185	uracil > GO:uracil transport ; GO:0015857 > GO:uracil transporter activity ; GO:0015210	2	**0.032**	2	0.058
4.10.31	chloramphenicol > GO:chloramphenicol transport ; GO:0042892	2	**0.023**	2	**0.022**
4.10.5	alkanesulfonate > GO:alkanesulphonate transport ; GO:0042918 > GO:alkanesulphonate transporter activity ; GO:0042959	3	0.159	3	**0.036**
4.10.78	glycerol-3-P > GO:glycerol transport ; GO:0015793 > GO:glycerol-3-phosphate transporter activity ; GO:0015169	5	**0.020**	5	0.103
4.10.79	glycine betaine choline transport > GO:betaine transport ; GO:0015838 > choline transport ; GO:0015871	5	**0.046**	5	0.325
4.2.1	Porters (Uni-, Sym- and Antiporters) > GO:symporter activity ; GO:0015293 > GO:antiporter activity ; GO:0015297 > GO:uniporter activity ; GO:0015292	203	**0.050**	203	0.178
4.2.1.20	The Inorganic Phosphate Transporter (PiT) Family > GO:inorganic phosphate transporter ; GO:0005315	3	**0.018**	3	0.454
4.2.1.21	The Solute:Sodium Symporter (SSS) Family > GO:solute:sodium symporter ; GO:0015370	4	**0.015**	4	**0.008**
4.2.1.7	The Small Multidrug Resistance (SMR) Family > GO:multidrug transporter ; GO:0015239	6	**0.050**	6	0.190
4.3.1	Pyrophosphate Bond (ATP; GTP; P2) Hydrolysis-driven Active Transporters > GO:ATPase activity ; GO:0016887	262	**0.046**	262	0.603
4.3.1.1	The ATP-binding Cassette (ABC) Superfamily + ABC-type Uptake Permeases > GO:ATP-binding cassette (ABC) transporter activity ; GO:0004009	235	**0.029**	235	0.417
4.8.1.3	MPA1 Family auxillary transport protein> GO:.	2	0.083	2	**0.035**
4.9.1	Recognized transporters of unknown biochemical mechanism > GO:transporter activity ; GO:0005215 > GO:transport ; GO:0006810	4	0.243	4	**0.034**

Significant (Wilcoxon rank sum test; *P* ≤ 0.05 shown in bold) functional categories involved in environmental shift evolutionary condition.

Bin	Functional annotation	No of Genes	MGGal/ MGAdpGal	No of Genes	DHGal/ DHAdpGal
1.2	Macromolecule degradation > GO:macromolecule catabolism ; GO:0009057	104	0.801	104	**0.022**
6.3	Surface antigens (ECA, O antigen of LPS) > GO:cell surface antigen activity, host-interacting ; GO:0042280	63	0.184	63	**0.002**
8	extrachromosomal > GO:.	292	0.233	292	**0.002**
8.1	Prophage genes and phage related functions > GO:.	258	0.459	258	**0.002**
8.4	Colicin related > GO:.	9	**0.028**	9	0.660
1.2.2	DNA > GO:DNA catabolism ; GO:0006308	25	0.854	25	**0.023**
1.2.4	Polysaccharides > GO:polysaccharide catabolism ; GO:0000272	7	**0.034**	7	0.978
1.5.1.11	Serine > GO:serine biosynthesis ; GO:0006564	6	**0.046**	6	0.600
1.5.1.16	Histidine > GO:histidine biosynthesis ; GO:0000105	12	**0.003**	12	0.845
1.5.3.8	Thiamine (Vitamin B1) > GO:thiamin biosynthesis ; GO:0009228	15	0.666	15	**0.013**
1.6.3	Lipopolysaccharide > GO:lipopolysaccharide biosynthesis ; GO:0009103	47	0.081	47	**0.001**
1.6.3.1	O antigen > GO:O antigen biosynthesis ; GO:0009243	15	**0.014**	15	**0.000**
1.6.3.2	Core region > GO:lipopolysaccharide core region biosynthesis ; GO:0009244	17	0.829	17	**0.004**
1.6.4	Enterobacterial common antigen (surface glycolipid) > GO:enterobacterial common antigen biosynthesis ; GO:0009246	12	0.053	12	**0.016**
1.7.10	Sugar nucleotide biosynthesis, conversions > GO:nucleotide-sugar biosynthesis ; GO:0009226	15	0.341	15	**0.008**
1.7.13	Amino acid conversion > GO:amino acid metabolism ; GO:0006520	3	**0.011**	3	0.770
1.7.14	Polyamine biosynthesis > GO:polyamine biosynthesis ; GO:0006596	19	**0.004**	19	0.710
1.7.7	Galactose metabolism > GO:galactose metabolism ; GO:0006012	5	**0.036**	5	0.286
2.2.5	tRNA > GO:tRNA metabolism ; GO:0006399	2	**0.036**	2	0.401
3.1.2.3	Repressor > GO:transcriptional repressor activity ; GO:0016564	138	**0.037**	138	0.509
3.1.4	Regulation level unknown > GO:.	73	**0.019**	73	0.746
3.3.1	Operon (regulation of one operon) > GO:.	127	0.205	127	**0.027**
4.10.118	methionine > GO:methionine transport ; GO:0015821 > GO:methionine transporter activity ; GO:0015191	4	0.904	4	**0.045**
4.10.12	amino acid > GO:amino acid transporter activity ; GO:0015171 > GO:amino acid transport ; GO:0006865	12	0.628	12	**0.047**
4.10.173	sugar > GO:carbohydrate transport ; GO:0008643 > GO:carbohydrate transporter activity ; GO:0015144	33	**0.006**	33	0.674
4.10.177	thiamine > GO:thiamin transport ; GO:0015888 > GO:thiamin transporter activity ; GO:0015234	4	0.920	4	**0.001**
4.10.191	Zn > GO:zinc ion transport ; GO:0006829 > GO:zinc ion transporter activity ; GO:0005385	3	**0.028**	3	0.491
4.10.71	glucose > GO:glucose transport ; GO:0015758 > GO:glucose transporter activity ; GO:0005355	2	0.099	2	**0.047**
4.10.82	H+ > GO:proton transport ; GO:0015992 > GO:hydrogen ion transporter activity ; GO:0015078	14	0.530	14	**0.007**

4.2.1.38	The K+ Transporter (Trk) Family > GO:potassium:hydrogen antiporter ; GO:0015386 > GO:potassium:hydrogen symporter ; GO:0015387	3	0.776	3	**0.039**
4.3.1.1.1	ATP binding component > GO:.	89	0.159	89	**0.018**
4.3.1.2	The H+/Na+-translocating F-, V- and A-type ATPase (F-ATPase) Superfamily > GO:hydrogen-translocating F-type ATPase complex (sensu Bacteria) ; GO:0045256 > GO:hydrogen-translocating V-type ATPase complex ; GO:0016471	8	0.640	8	**0.012**

Significant (Wilcoxon rank sum test; *P* ≤ 0.05 shown in bold) functional categories involved in prolonged stationary phase evolutionary condition.

**Bin**	**Functional annotation**	**No of Genes**	**MG/ MGStat**	**No of Genes**	**DH/ DHStat**
1.6	Macromolecules (cellular constituent) biosynthesis > GO:macromolecule biosynthesis ; GO:0009059	272	**0.001**	272	0.335
5.2	Cell cycle physiology > GO:cell cycle ; GO:0007049	5	0.786	5	**0.009**
5.3	Motility (incl. chemotaxis, energytaxis, aerotaxis, redoxtaxis) > GO:taxis ; GO:0042330	59	**0.043**	59	0.946
6.3	Surface antigens (ECA, O antigen of LPS) > GO:cell surface antigen activity, host-interacting ; GO:0042280	65	**0.048**	65	0.280
6.6	Ribosome > GO:cytosolic ribosome (sensu Bacteria) ; GO:0009281	66	0.514	66	**0.031**
8	extrachromosomal > GO:.	298	0.214	298	**0.001**
8.1	Prophage genes and phage related functions > GO:.	264	0.107	264	**0.000**
1.1.1.18	Trehalose degradation, low osmolarity > GO:trehalose catabolism ; GO:0005993	3	0.898	3	**0.047**
1.1.1.25	L-ascorbate degradation > GO:L-ascorbic acid catabolism ; GO:0019854	11	0.981	11	**0.001**
1.1.1.3	D-arabinose catabolism > GO:D-arabinose catabolism ; GO:0019571	3	0.077	3	**0.011**
1.1.2.2	3-phenylpropionate and 3-(3-hydroxyphenyl)propionate degradation > GO:3-phenylpropionate catabolism ; GO:0019380 > GO:3-(3-hydroxy)phenylpropionate catabolism ; GO:0019622	11	0.527	11	**0.018**
1.1.2.3	Propionate degradation > GO:propionate catabolism ; GO:0019543	9	0.968	9	**0.010**
1.1.3.7	Threonine catabolism > GO:threonine catabolism ; GO:0006567	12	**0.050**	12	**0.023**
1.1.4.3	Ornithine degradation > GO:ornithine catabolism ; GO:0006593	2	**0.026**	2	0.104
1.1.5	Others > GO:.	29	0.171	29	**0.033**
1.1.5.2	Ethanol degradation > GO:ethanol catabolism ; GO:0006068	10	0.347	10	**0.018**
1.5.1.17	Alanine > GO:alanine biosynthesis ; GO:0006523	4	0.962	4	**0.004**
1.5.1.21	Homoserine > GO:homoserine biosynthesis ; GO:0009090	6	0.271	6	**0.024**
1.5.1.6	Asparagine > GO:asparagine biosynthesis ; GO:0006529	4	**0.038**	4	0.191
1.5.3	Cofactor, small molecule carrier > GO:coenzymes and prosthetic group biosynthesis ; GO:0046138	168	**0.009**	168	0.300
1.5.3.12	Heme, porphyrine > GO:heme biosynthesis ; GO:0006783 > GO:porphyrin biosynthesis ; GO:0006779	16	0.971	16	**0.041**

1.5.3.2	Folic acid > GO:folic acid biosynthesis ; GO:0046656	10	**0.035**	10	0.061
1.5.3.5	Coenzyme A > GO:coenzyme A biosynthesis ; GO:0015937	11	**0.038**	11	0.467
1.5.3.7	Nicotinamide adenine dinucleotide (NAD) > GO:nicotinamide adenine dinucleotide biosynthesis ; GO:0009435	15	0.142	15	**0.025**
1.5.4	Fatty acid and phosphatidic acid > GO:fatty acid biosynthesis ; GO:0006633 ; GO:phosphatidic acid biosynthesis ; GO:0006654	27	0.319	27	**0.020**
1.6.1	Phospholipid > GO:phospholipid biosynthesis ; GO:0008654	21	**0.000**	21	0.142
1.7.26	Allantoin assimilation > GO:allantoin assimilation ; GO:0009442	8	0.722	8	**0.018**
1.7.8	Gluconeogenesis > GO:gluconeogenesis ; GO:0006094	17	0.056	17	**0.050**
2.3.2	Translation > GO:protein biosynthesis ; GO:0006412	73	0.543	73	**0.021**
2.3.8	Ribosomal proteins > GO:structural constituent of ribosome ; GO:0003735	56	0.241	56	**0.028**
3.1.2.1	Sigma factors, anti-sigmafactors > GO:sigma factor activity ; GO:0016987 > GO:sigma factor antagonist activity ; GO:0016989	17	**0.004**	17	0.132
4.1.2	Beta barrel porins (The Outer Membrane Porin (OMP) Functional Superfamily) > GO:porin ; GO:0015288	18	**0.045**	18	0.570
4.10.10	allose/ribose > GO:allose transport ; GO:0015754 > GO:ribose transport ; GO:0015752 > GO:allose transporter activity ; GO:0015593 > GO:ribose transporter activity ; GO:0015591	4	**0.039**	4	**0.003**
4.10.118	methionine > GO:methionine transport ; GO:0015821 > GO:methionine transporter activity ; GO:0015191	4	**0.010**	4	0.118
4.10.119	methylgalactoside/galactose > GO:galactose transporter activity ; GO:0005354 > GO:galactose transporter activity ; GO:0005354 > GO:methylgalactoside transporter activity ; GO:0015592 > GO:methylgalactoside transport ; GO:0015765	4	0.462	4	**0.016**
4.10.143	Ni++ > GO:nickel ion transport ; GO:0015675 > GO:nickel ion transporter activity ; GO:0015099	5	0.343	5	**0.032**
4.10.148	oligopeptide > GO:oligopeptide transport ; GO:0006857 > GO:oligopeptide transporter activity ; GO:0015198	12	0.557	12	**0.001**
4.10.152	peptide > GO:peptide transport ; GO:0015833 > GO:peptide transporter activity ; GO:0015197	12	**0.025**	12	0.060
4.10.155	phosphate > GO:phosphate transport ; GO:0006817 > GO:phosphate transporter activity ; GO:0015114	7	0.277	7	**0.006**
4.10.185	uracil > GO:uracil transport ; GO:0015857 > GO:uracil transporter activity ; GO:0015210	2	0.832	2	**0.034**
4.10.29	cellobiose/arbutin/salicin > GO:salicin transport ; GO:0042948 > GO:salicin transporter activity ; GO:0042950 > GO:cellobiose transport ; GO:0019533 > GO:cellobiose transporter activity ; GO:0019191 > GO:salicin transporter activity ; GO:0042950 > GO:salicin transporter activity ; GO:0042950	4	**0.004**	4	**0.027**
4.10.48	D-ribose > GO:ribose transport ; GO:0015752 > GO:D-ribose transporter activity ; GO:0015591	6	**0.042**	6	0.469
4.10.54	Fe++ > GO:ferric iron transport ; GO:0015682 > GO:ferric iron transporter activity ; GO:0015091	2	**0.025**	2	0.955
4.10.58	ferrichrome > GO:ferrichrome transport ; GO:0042928 > GO:ferrichrome transporter activity ; GO:0042929	5	0.581	5	**0.017**
4.10.75	glutamate/aspartate > GO:glutamate transport ; GO:0015813 > GO:aspartate transport ; GO:0015810 > GO:glutamate transporter activity ; GO:0005313 > GO:aspartate transporter activity ; GO:0015183	4	0.789	4	**0.008**
4.10.78	glycerol-3-P > GO:glycerol transport ; GO:0015793 > GO:glycerol-3-phosphate transporter activity ; GO:0015169	5	0.256	5	**0.033**
4.10.82	H+ > GO:proton transport ; GO:0015992 > GO:hydrogen ion transporter activity ; GO:0015078	14	0.971	14	**0.022**
4.2.1.13	The C4-Dicarboxylate Uptake (Dcu) Family > GO:C4-dicarboxylate transporter ; GO:0015556	2	**0.045**	2	0.209
4.2.1.21	The Solute:Sodium Symporter (SSS) Family > GO:solute:sodium symporter ; GO:0015370	4	0.848	4	**0.020**
4.2.1.40	The Nucleobase:Cation Symporter-2 (NCS2) Family > GO:nucleobase:cation symporter ; GO:0015391	5	0.879	5	**0.006**

4.2.1.42	The Aromatic Amino Acid Permease (ArAAP) Family > GO:hydroxy/aromatic amino-acid permease ; GO:0015507	7	**0.001**	7	0.946
4.2.1.47	The Divalent Anion:Na+ Symporter (DASS) Family > GO:.	3	**0.048**	3	0.153
4.3.1.1.1	ATP binding component > GO:.	92	**0.005**	92	0.068
4.3.1.1.3	periplasmic binding component> GO:.	51	0.428	51	**0.041**
4.4.1.3	PTS Lactose-N,N'-Diacetylchitobiose-betaucoside (Lac) Family > GO:.	3	**0.018**	3	**0.016**
4.4.1.5	The PTS Galactitol (Gat) Family > GO:galactitol permease ; GO:0015588	5	**0.021**	5	0.710
4.8.1.3	MPA1 Family auxillary transport protein> GO:.	2	0.151	2	**0.035**
4.8.1.7	The Phosphotransferase System Enzyme I (EI) Family> GO:.	7	0.214	7	**0.030**
4.9.2.22	The Putative Permease (PerM) Family> GO:.	2	0.680	2	**0.034**
5.5.4	pH response > GO:response to pH ; GO:0009268	13	0.394	13	**0.033**
5.5.6	Other stresses (mechanical, nutritional, oxidative) > GO:response to mechanical stimulus ; GO:0009612 > GO:response to nutrients ; GO:0007584 > GO:response to oxidative stress ; GO:0006979	22	0.353	22	**0.025**
